# A Pilot Randomized, Double-Blind, Placebo-Controlled Parallel Group Trial Evaluating the Effect of 8 Week-Consumption of Guava Jelly Drink in Improving Cognition and Mental Well-Being in Working-Age Adults

**DOI:** 10.3390/foods15142461

**Published:** 2026-07-11

**Authors:** Hai-Ha Nguyen, Jintanaporn Wattanathorn, Wipawee Thukham-Mee, Supaporn Muchimapura, Pongsatorn Paholpak

**Affiliations:** 1 Department of Physiology and Graduate School (Neuroscience Program), Faculty of Medicine, Khon Kaen University, Khon Kaen 40002, Thailand; nhaiha@ctump.edu.vn; 2 Department of Physiology, Faculty of Medicine, Khon Kaen University, Khon Kaen 40002, Thailand; meewep@gmail.com (W.T.-M.); supmuc@kku.ac.th (S.M.); 3 Research Institute for High Human Performance and Health Promotion, Khon Kaen University, Khon Kaen 40002, Thailand; 4 Department of Psychiatry, Faculty of Medicine, Khon Kaen University, Khon Kaen 40002, Thailand; ppaholpak@kku.ac.th

**Keywords:** guava jelly drink, randomized controlled trial, working-aged adults, working memory, selective attention, perceived stress, anxiety

## Abstract

Given the lack of clinical data supporting the effects of a novel guava jelly drink on cognition and mood regulation, we aimed to explore these effects and their possible mechanisms in working-age volunteers. In an 8-week, three-arm double-blind, placebo-controlled trial, healthy males and females aged 20–40 years old (N = 25/arm) were randomly assigned to consume 86 g per day of either a placebo or guava jelly drink containing either a low (36.6%) or high (73.2%) dose of guava juice with mint syrup. N100 and P300 brain waves, working memory, Perceived Stress Scale (PSS), Hospital Anxiety and Depression Scale (HADS), and biomarkers related to oxidative stress, inflammation, neurotransmitters, and gut microbiota were assessed at baseline and after 4 and 8 weeks of consumption. The low-dose group showed improvements in N100 amplitude, P300 latency, and HADS anxiety score, whereas the high-dose group exhibited improved N100 amplitude, working memory, PSS score, and total HADS score. In addition, the high-dose group also exhibited increased GPx activity without a reduction in MDA. Overall, these results suggest that guava jelly drink positively modulates perceived stress, anxiety, selective attention, and working memory. However, the precise underlying mechanism requires further study.

## 1. Introduction

Currently, approximately 60% of the global population is engaged in work [1], and more than half are exposed to poor working environments, including excessive workloads, job insecurity, and financial strain. Job and general stresses can contribute to mental disorders, such as anxiety and depression [2]. A recent meta-analysis revealed that stress, depression, and anxiety affect 25.2%, 28.2%, and 29.6% of the general population, respectively [3]. Moreover, new data released by the World Health Organization (WHO) demonstrate that in 2025, more than 1 billion people worldwide live with mental disorders, particularly anxiety and depression [4]. These conditions have a substantial negative impact on the global economy. It has been estimated that the annual global productivity loss associated with anxiety and depression is approximately 1 trillion USD [4]. Beyond the mental disorders mentioned above, stress exposure, particularly repetitive stress exposure, is also associated with cognitive decline [5,6], particularly in working memory [7], processing speed [8], and attention [9]. Stress-related cognitive impairment not only reduces quality of life (QOL) but also increases the risk of Alzheimer’s disease [10], which, in turn, contributes to high annual healthcare costs [11]. At present, stress exposure is increasing worldwide and appears to be an avoidable phenomenon. Therefore, the prevalence of stress-related mental health disorders and cognitive impairment is also increasing, highlighting the need for prioritized management. Owing to their increasing prevalence and high negative impacts, efforts to promote cognitive enhancement and prevent mental health disorders have become a growing priority, especially for working-age individuals who play an important role in economic productivity.

At present, numerous drugs are available for the treatment of anxiety, depression, and cognitive impairment, but most are associated with side effects and poor consumer compliance. Additionally, consumers increasingly prefer natural ingredients over chemically synthesized compounds such as pharmaceutical drugs [12]. Moreover, the medical paradigm has shifted from a therapeutic strategy to a preventive strategy because prevention is more effective in improving long-term outcomes and reducing costs. These factors have driven the increasing demand in functional foods that may help prevent mental health disorders and cognitive impairment. Accumulating evidence demonstrates that oxidative stress and inflammation are implicated in the pathophysiology of mental health and cognition disorders [13,14,15,16,17]. Therefore, substances possessing antioxidant and anti-inflammation effects, such as polyphenol and vitamin C, may be beneficial [18,19].

*Psidium guajava* L. (guava) is a tropical fruit that is rich in polyphenols and vitamin C. However, fruit composition appears to depend on various factors, such as maturity and environmental conditions [20]. To increase the feasibility and efficiency for controlling potential active ingredients and health benefits, while also adding value, the development of innovative functional food has gained attention. Recently, we developed a novel functional drink from unripe pink guava (Fen Hong Mi cultivar) that is rich in total phenolic compound (4.45 mg GAE/g), flavonoid (0.69 mg quercetin/g), and vitamin C levels (44.76 mg/100 g). The main flavonoids found in the functional drink are gallic acid (0.6 mg/100 g sample), kaempferol (0.3 mg/100 g sample), and quercetin (0.1 mg/100 g sample) [21]. In addition, the product also contains approximately 96 µg/100 g beta-carotene. This drink exhibits antioxidant and anti-inflammation activities. In addition, it also improves the availability of neurotransmitters that play pivotal roles in the regulation of mood and cognition. Therefore, this drink exhibits the potential to improve cognition and mental health. However, clinical evidence regarding this potential has not been available to date. Therefore, we aimed to evaluate the effect of 8-week consumption of pink guava jelly drink on anxiety, depression, stress perception, and cognitive function in working-age volunteers. Furthermore, possible underlying mechanisms and adverse events were also explored.

## 2. Materials and Methods

### 2.1. Preparation of the Guava Based-Jelly Drink

Unripe pink guava fruits were obtained from Tanaporn Garden, Nonthaburi, Thailand, during the period from July to October 2024. The method used to prepare the guava jelly drink was mentioned in detail elsewhere [21]. In brief, small pieces of guava fruit pulps were blended with water at a ratio of 1 kg to 1 L (*w*/*v*) and then boiled for 20 min. The obtained guava juice was filtered and mixed with mint syrup and pomelo peel-derived dietary fiber. The juice mixture was heated with continuous stirring for 15 min or until complete dissolution occurred. Then, the juice was mixed with guava jelly at a ratio of 10:3 (*w*/*w*). For the low-dose formulation, half the quantity of guava juice and mint syrup were used. The placebo jelly drink was prepared using the same procedure, except that guava juice was omitted and honey syrup was replaced with mint syrup.

Dose calculation was performed based on the assumption of a one-compartment human distribution volume and the EC_50_ (half-maximal effective concentration) obtained from an in vitro assessment of the acetylcholinesterase inhibitory (AChEI) activity of the developed guava jelly drink. According to this study, we selected AChEI as the primary outcome for the dose calculation because acetylcholinesterase inhibitors (AChEIs) are the current first-line treatment for mild-to-moderate Alzheimer’s disease and other dementias [22,23]. According to our previous in vitro study, the EC_50_ for in vitro AChE inhibitory activity was 17.2 mg/mL [21], and the estimated human blood volume was 5 L. Therefore, the estimated dose of the guava jelly drink required for the half-maximal effective concentration of AChEI was approximately 86 g (73.2% guava juice with mint syrup). This dose served as the high dose in this study, and the low dose in this study was derived as half of the concentration of the high dose, corresponding to the guava jelly drink containing 36.6% guava juice with mint syrup. The ingredients of the placebo, low-dose, and high-dose guava jelly drinks are shown in Table 1. All the tested products in this study were identical in texture, color, odor, and packaging. The participants obtained the assigned product according to the schedule from an independent staff member of the research center who was not actively involved in the study. Therefore, the researchers who were actively involved in this study and the participants were blinded to the type of beverage the volunteers consumed.

### 2.2. Research Ethics and Regulatory Approval

This study was a pilot study conducted as an 8-week double-blind and placebo-controlled randomized trial. All experiments were conducted at the Research Institute for Human High Performance and Health Promotion (HHP&HP), Faculty of Medicine, Khon Kaen University. This clinical trial was registered at Thaiclinicaltrials.org (TCTR20230418003) and was performed in full compliance with the International Conference on Harmonisation (ICH) guidelines for Good Clinical Practice (GCP), as well as the ethical principles set forth in the Declaration of Helsinki and its subsequent revisions. This study was performed with the approval of the Ethical Committee of the Faculty of Medicine, Khon Kaen University (HE661153). Written informed consent was obtained from all participants before the initiation of the trial.

### 2.3. Subjects

The sample size calculation was performed based on our primary outcome, namely, cognitive performance derived from event-related potential (ERP) [24]. At a two-sided significance level of 5% and a statistical power of 80%, 20 participants per group were required. After accounting for an expected dropout rate of 20%, the final sample size was determined to be 25 participants per group, yielding a total of 75 participants. A total of 79 healthy men and women from the Northeastern region of Thailand were recruited for the study using advertisements. Eligible individuals at an age between 20 and 40 years old with a body mass index (BMI) ranging from 18.5 to 25 kg/m^2^ were included in this study. All participants were required to abstain from consuming tea, coffee, chocolate, and alcohol for at least 12 h prior to and throughout the study period. Individuals were excluded if they were undergoing treatment with any medications or consuming herbal products that could affect nervous system function, either during the trial or within 3 months prior to its commencement. Additional exclusion criteria included a history or diagnosis of cancer, diabetes, cardiovascular disease, respiratory or gastrointestinal disorders, traumatic brain injury, neurological or psychiatric conditions, or known allergies. Volunteers with moderate to severe anxiety or depression, as indicated by a Hospital Anxiety and Depression Scale (HADS) score of 11 or higher, were also excluded. Pregnant or breastfeeding individuals, those with a history of alcohol addiction, smokers consuming more than 10 cigarettes per day or unable to abstain from smoking during the study, and those who had taken probiotics within the past 3 months or antibiotics within the past week were not eligible to participate. Furthermore, individuals who engaged in physical exercise more than three times per week were excluded from the trial.

After the screening process, which involved an interview and physical examination, four individuals were excluded: one due to elevated gamma-glutamyl transferase (GGT) levels, one due to elevated amylase levels, and two due to BMI values above the eligible range. A total of 75 eligible participants were randomized and allocated to one of the following groups, including (1) placebo, (2) low-dose guava jelly, and (3) high-dose guava jelly, at a ratio of 1:1:1 (N = 25/group). Randomization was performed by an independent staff member of the research center using a computer-generated block randomization design with a block size of 6. The principal investigator was provided a randomization code in a concealed envelope, and allocation concealment was maintained until the analysis was completed. In this study, participants were considered compliant if they returned at least 90% of the guava jelly drink packages after each visit. Compliance was assessed at every visit by counting the used guava jelly drink packages that were returned at each visit.

### 2.4. Study Design

**Figure 1:**
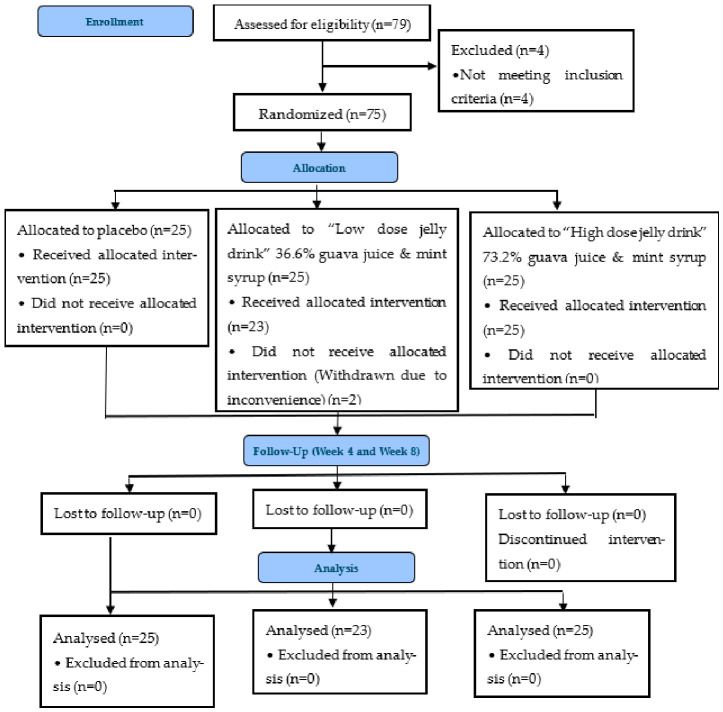
Flow diagram of study design.

The current study was a pilot, 3-arm randomization, double bind, parallel-group controlled trial. A total of 79 healthy individuals were recruited using advertisements at the Faculty of Medicine, Khon Kaen University. After the interview and physical examination, 75 eligible participants were randomly allocated to one of the following arms: placebo, low-dose guava jelly drink, and high dose guava jelly drink. The treatment was performed for 8 weeks. The primary outcome was cognition assessed using the N100 and P300 wave components derived from event-related potential (ERP) as indicators. The secondary outcomes comprised working memory, assessing using a computerized battery test; mental health-related parameters, assessed using the Perceived Stress Scale (PSS-10) and the Hospital Anxiety and Depression Scale (HADS); serum activities of enzymes involved in the inactivation of key neurotransmitters involved in cognition and mood regulation, including acetylcholinesterase (AChE), total monoamine oxidase (MAO), monoamine oxidase A (MAO-A), monoamine oxidase B (MAO-B), gamma-aminobutyric acid transaminase (GABA-T), and glutamate decarboxylase (GAD), as well as serum levels of cortisol, brain-derived neurotrophic factor (BDNF), and pro-inflammatory cytokines such as interleukin-1 beta (IL-1β), interleukin-6 (IL-6), and tumor necrosis factor-alpha (TNF-α). Oxidative stress biomarkers, including malondialdehyde (MDA), and antioxidant activities, such as catalase (CAT) and glutathione peroxidase (GPx), were also assessed. In addition, densities of lactic acid-producing bacteria (LAB), *Lactobacillus* spp., and *Bifidobacterium* spp. were also measured to assess gut microbiota modification. To assess consumption safety, hematological and clinical chemistry parameters were measured together with adverse events. All primary and secondary outcomes were assessed at baseline and 4 and 8 weeks during the study period, except for the assessments of fecal densities of LAB, *Lactobacillus* spp., and *Bifidobacterium* spp., which were conducted only at baseline and 8 weeks. During the trial, all volunteers received instructions not to consume any additional functional foods or beverages and to maintain their usual lifestyles. Compliance was evaluated by counting the functional jelly drink packages returned at each visit. An overview of the study protocol is illustrated in Figure 1. In this study, the physical examination was performed by a physician, and all processes were handled by the project physician and trained staff.

### 2.5. Event-Related Potentials (ERP)

Event-related potentials (ERPs) are electrical potential responses to specific stimuli and are commonly used in neuroscience research to assess cognitive function. The classical “oddball paradigm” of auditory ERP was used to assess the cognitive function of all participants using the amplitudes and latencies of N100, the first negative peak with a latency of approximately 100 ms [25], and P300, a positive peak with a latency around 300 ms, as indicators [26]. Brain activity was recorded using a 40-channel Ag-AgCl electrode cap based on the international 10–20 system, with connected mastoids acting as the reference electrode [27] (Neuroscan, Inc., Sterling, FL, USA). Participants were required to wear headphones and listen to a series of target and non-target stimuli. To respond to the auditory stimuli, participants press the corresponding button for either target or non-target tones. All volunteers were instructed to focus on and count high-frequency stimuli [28]. The latency and maximum amplitudes of the N100 and P300 waveforms were measured at the Cz and Fz locations, where the maximum changes were observed. The continuous EEG signal was digitized at a sampling rate of 1000 Hz with an analog filter band pass of 1–100 Hz. All artifacts, including ocular artifacts, were removed from the continuous EEG prior to the extraction of ERP waves. Artifact-free EEG epochs were extracted from the 100 msec before stimulus onset to 500 msec post-stimulation. Baseline correction was also applied to each epoch. Any changes in voltage below 0.1 μV or above 50 μV were not included in the analysis.

### 2.6. Computerized Battery Test

In this study, working memory was evaluated using computerized battery test tasks, including digit vigilance, choice reaction time, simple reaction time, inhibition, spatial working memory, picture recognition, and word recognition, to evaluate multiple cognitive domains, including attention, processing speed, inhibitory control, working memory, and recognition memory [29,30,31,32,33]. Task performance was reported as reaction time (minutes). Fluency, which was defined as the number of correct responses per minute, was indicated as task efficiency. Higher fluency scores indicated better cognitive performance.

#### 2.6.1. Choice Reaction Time

Series of “yes” or “no” words were displayed on the screen, and the participants must press the corresponding button as fast as possible.

#### 2.6.2. Delayed Word Recognition

Fifteen original words were displayed randomly with 15 distractor words simultaneously. The participants must identify the words that belonged to the original words and press the corresponding “yes” or “no” buttons on the keyboard as quickly as possible.

#### 2.6.3. Picture Recognition

A set of 15 photographic pictures was provided to each individual at a frequency of one picture every 3 s, and the duration between the stimuli was 1 s. A set of original pictures was displayed randomly with 15 distractor pictures simultaneously. The participants must identify the pictures that belonged to the original pictures and press the corresponding “yes” or “no” buttons on the keyboard as quickly as possible.

#### 2.6.4. Simple Reaction Time

A series of circles appeared on the screen at a duration of 2 s and interstimulus durations ranging from 2 to 3.5 s. Each individual must press the “yes” button as fast as possible.

#### 2.6.5. Digit Vigilance

Each subject must press the “yes” button as quickly as possible when a target three-digit number constantly presented on the right side of the screen at a speed of 60 numbers per minute matched a series of three-digit numbers that were provided on the left side of the screen.

#### 2.6.6. Inhibition

A circle or double circles appeared on the screen for 2 s. If a circle appeared, the participants had to press “yes” as fast as possible. If double circles appeared, they did nothing. There were 50 trials with random intervals between the stimuli ranging from 2 to 3 s.

#### 2.6.7. Spatial Working Memory

A yellow window image appeared for 3 s on the screen within any cell of a 4 × 4 table, and it disappeared for 2 s before it reappeared in another cell. This loop was repeated, and the participant had to memorize the cells in which the yellow windows appeared. Then, a series of window images with various colors appeared one by one in any cell for 3 s. During this time, the participant had to evaluate whether the position corresponded to the initial yellow window that appeared by pressing the “yes” or “no” button as fast as possible.

### 2.7. Mental Health Assessment

Stress was assessed using the Perceived Stress Scale (PSS-10), a widely used self-report questionnaire, that measures the degree to which individuals perceive their lives as stressful, focusing on unpredictability, uncontrollability, and overload. This test focused on subjective feelings of stress and the perceived ability to cope with stressors [34,35]. It comprises 10 items, and each item is rated on a 5-point Likert scale from 0 (never) to 4 (very often). The higher scores indicate higher perceived stress.

Anxiety and depression were assessed using the Hospital Anxiety and Depression Scale (HADS), a validated 14-item self-report tool. It comprises 14 items, including seven items for the anxiety subscale (HADS Anxiety) and seven items for the depression subscale (HADS Depression). Each item is scored from 0 (not at all) to 3 (most of the time). The overall score ranges from 0 to 21, with higher values corresponding to increased severity [36,37].

### 2.8. Blood Collection and Serum Preparation

Venous blood was collected after at least 8 h of fasting. Following collection, the blood was placed into tubes containing a clot activator and allowed to stand at room temperature for 30 min to complete coagulation. Then, the samples were centrifuged at 2000× *g* for 10 min at room temperature to obtain serum. The supernatant was promptly stored at −80 °C within 1 h of collection for subsequent analyses.

### 2.9. Neurotransmitters Assessment

In this study, neurotransmitters were indirectly assessed by evaluating the activities of enzymes that play important roles in their inactivation pathways.

#### 2.9.1. Determination of Acetylcholinesterase (AChE) Activity

Acetylcholine was indirectly assessed by measuring acetylcholinesterase (AChE) activity with a colorimetric method [32,33]. In brief, a reaction mixture containing 10 µL of 0.2 M 5,5′-dithio-bis-2-nitrobenzoic acid (DTNB) (Sigma-Aldrich, St. Louis, MO, USA) and 200 µL of 0.2 M (pH 8.0) phosphate-buffered saline (PBS) was mixed with 10 µL of sample and subjected to a 5 min incubation at room temperature. Following this step, absorbance was measured at 415 nm before and after adding 10 µL of 30 mM acetyl thiocholine iodide (AChID) (Sigma-Aldrich, St. Louis, MO, USA). The AChE activity results were reported as nmol/mg protein.

#### 2.9.2. Determination of Monoamine Oxidase (MAO), Monoamine Oxidase A (MAO-A) and Monoamine Oxidase B (MAO-B) Activities

Monoamine transmitters were measured by assessing the activities of MAO, MAO-A, and MAO-B with a colorimetric method [24]. The chromogenic solution was generated by combining 1 mM vanillic acid (Sigma-Aldrich, St. Louis, MO, USA), 500 µM 4-aminoantipyrine (Sigma-Aldrich, St. Louis, MO, USA), and peroxidase (4 U/mL) (Sigma-Aldrich, St. Louis, MO, USA) in 0.2 M PBS (pH 7.6). To determine MAO activity, the mixture, including 50 µL of sample, 50 µL of chromogenic solution, and 200 µL of 500 µM P-Tyramine (Sigma-Aldrich, St. Louis, MO, USA), was prepared and incubated at 37 °C for 30 min. The absorbance at 490 nm was recorded at the end of the incubation.

MAO-A inhibitors are beneficial for improving anxiety and depression, whereas MAO-B inhibition appears to be effective for improving cognitive function [38]. To determine MAO-A activity, 50 µL of sample was added to a reaction mixture including 50 µL of chromogenic solution and 50 µL of 500 nM pargyline (Sigma-Aldrich, St. Louis, MO, USA) and kept at 37 °C for 30 min. Then, 150 µL of 500 µM P-tyramine (Sigma-Aldrich, St. Louis, MO, USA) was added, and the absorbance at 490 nm was measured at the end of the incubation. MAO-B activity was determined using the same experimental procedure, except that pargyline was replaced with clorgyline. All MAO, MAO-A, and MAO-B activity results were reported as μmol/mg protein.

#### 2.9.3. Determination of Gamma-Aminobutyric Acid-Transaminase (GABA-T) Activity

The activity of GABA-T, an enzyme that inactivates gamma-aminobutyric acid (GABA), was assessed as an indirect indicator of GABA. Briefly, a sample aliquot at a volume of 100 μL was mixed with 200 μL of the mixture containing 20 mM GABA (Sigma-Aldrich, St. Louis, MO, USA), 10 mM α-ketoglutarate (Sigma-Aldrich, St. Louis, MO, USA), and 0.5 mM NAD (Sigma-Aldrich, St. Louis, MO, USA) in 0.05 M PBS pH 8.0. Then, the mixture was incubated at 30 °C for 30 min. At the end of the incubation period, the absorbance at 340 nm was measured [32]. GABA-T activity results were reported as nmol/mg protein.

#### 2.9.4. Determination of Glutamate Decarboxylase (GAD) Activity

Measurement of GAD, a crucial enzyme that irreversibly catalyzes the conversion of the excitatory neurotransmitter glutamate into GABA, was performed using an ELISA kit test (MBS261840, MyBioSource, San Diego, CA, USA) according to the company’s guidelines. In brief, 100 µL of sample was added to the wells and incubated at 37 °C for 90 min. After two washes with washing buffer, 100 µL of the biotinylated antibody was added and incubated at 37 °C for 60 min. The plate was then washed three times before adding 100 µL of the enzyme conjugate, followed by a 30 min incubation at 37 °C. After washing five times, 100 µL of the color reagent solution was added and incubated at 37 °C for 30 min. Finally, 100 µL of color reagent C was added. The solution was mixed thoroughly, and the absorbance was measured at 450 nm within 10 min [39].

### 2.10. Oxidative Stress Markers Assessment

Malondialdehyde (MDA), a marker of lipid peroxidation, was quantified in serum using the thiobarbituric acid reactive substances assay [40]. Briefly, a reaction mixture containing 50 μL of 8.1% sodium dodecyl sulfate (Sigma-Aldrich, St. Louis, MO, USA), 375 μL of 20% acetic acid (Sigma-Aldrich, St. Louis, MO, USA), 375 μL of 0.8% thiobarbituric acid (Sigma-Aldrich, St. Louis, MO, USA), and 150 μL of distilled water was combined with 50 μL of serum and incubated in boiling water at 95 °C for 1 h. After cooling at room temperature, 250 μL of distilled water and 1250 μL of an n-butanol:pyridine mixture (15:1) (Merck, Darmstadt, Germany) were added and thoroughly mixed. The samples were then centrifuged at 4000 rpm for 10 min, and the upper butanol phase was collected for measurement of absorbance at 532 nm. A standard curve was generated using 1,1,3,3-tetramethoxypropane (Sigma-Aldrich, St. Louis, MO, USA) at concentrations ranging from 0 to 15 μM. Malondialdehyde (MDA) levels were expressed as μmol/mg protein.

The activities of catalase (CAT) and glutathione peroxidase (GPx), two key enzymes responsible for scavenging reactive oxygen species, were measured to evaluate the antioxidant defense capacity under oxidative stress. Catalase (CAT) activity was determined by mixing 10 μL of serum with a reaction solution containing 50 μL of 30 mM H_2_O_2_ in 50 mM phosphate buffer (pH 7.0) (BDH Chemicals Ltd., London, UK), 25 μL of H_2_SO_4_ (Sigma-Aldrich, St. Louis, MO, USA), and 150 μL of 5 mM KMnO_4_ (Sigma-Aldrich, St. Louis, MO, USA), followed by measurement of absorbance at 490 nm. Here, CAT enzymes (Sigma-Aldrich, St. Louis, MO, USA) were used for the standard curve (0–100 U/mL) and expressed as units/mg protein [41].

For GPx activity measurement, 20 μL of sample was mixed with 10 μL of 1 mM dithiothreitol (Sigma-Aldrich, St. Louis, MO, USA) and 100 μL of 1 mM sodium azide (Sigma-Aldrich, St. Louis, MO, USA) in a 6.67 mM potassium phosphate buffer (Sigma-Aldrich, St. Louis, MO, USA) (pH 7), 10 μL of 50 mM glutathione (Sigma-Aldrich, St. Louis, MO, USA), and 100 μL of 30% hydrogen peroxide (BDH Chemicals Ltd., London, UK). After a 5 min incubation at room temperature, 10 μL of 10 mM DTNB (Sigma-Aldrich, St. Louis, MO, USA) was added, and absorbance was read at 412 nm. GSH-Px activity was calculated using a standard curve (1–50 U/mL) generated with GPx enzyme (Sigma-Aldrich, St. Louis, MO, USA) and expressed as units/mg protein [41].

### 2.11. Assessment of Inflammatory (TNF-α, IL-6, IL-1β), Stress-Related (Cortisol), and Neurotrophic (BDNF) Biomarkers

Serum cortisol levels, hematological changes, and blood biochemistry parameters were monitored at the Clinical Laboratory Unit of Srinagarind Hospital, Faculty of Medicine, Khon Kaen University.

TNF-α (ab108908, Abcam, Cambridge, UK) and IL-6 (ab178013, Abcam, Cambridge, UK) measurements were performed using an ELISA kit test according to the company’s guidelines as described in our previous study [32].

BDNF (ab212166, Abcam, Cambridge, UK) and IL-1β (ab214025, Abcam, Cambridge, UK) measurements were performed using an ELISA kit test according to the company’s guidelines. The procedure described below was applied for both assays. Briefly, 50 µL of sample was added to the wells, followed by 50 µL of the antibody cocktail. The plate was incubated at room temperature for 2 h on a shaker. After three washes with 1× wash buffer PT, 100 µL of 3,3′,5,5′-tetramethylbenzidine (TMB) solution was added and incubated for 10 min in the dark. The reaction was stopped with 100 µL of stop solution and mixed for 1 min. Then, the absorbance was read at 450 nm using an ELISA reader (Sunrise™, Tecan Trading AG, Männedorf, Switzerland) [42].

### 2.12. Determination of Total Phenolic Compounds in Serum

The total phenolic content in serum was determined using the Folin–Ciocalteu method [31]. Briefly, 20 μL of sample was mixed with Folin–Ciocalteu reagent (Sigma-Aldrich, St. Louis, MO, USA) and distilled water and incubated for 5 min. This was followed by the addition of 20% sodium carbonate (Sigma-Aldrich, St. Louis, MO, USA) and a further 2 h incubation at room temperature. Absorbance was measured at 765 nm, and results were expressed as mg gallic acid equivalents per milliliter of serum.

### 2.13. Determination of Lactic Acid-Producing Bacteria (LAB), Lactobacillus spp., and Bifidobacterium spp.

Following serial dilution of the samples, 0.1 mL aliquots from dilutions ranging from 10^−3^ to 10^−6^ were plated onto de Man–Rogosa–Sharpe (MRS) agar and HiCrome Bifidobacterium Agar (HiMedia Laboratories LLC, Kennett Square, PA, USA). The plates were incubated anaerobically at 37 °C for 48 h. The total number of LAB was quantified using a colony-counting machine. Subsequently, *Lactobacillus* spp. and *Bifidobacterium* spp. were differentiated based on colony morphology and Gram staining characteristics [32]. The results were presented in terms of log-transformed colony-forming units per milliliter (log CFU/mL).

### 2.14. Safety and Adverse Effect Assessment

To evaluate the safety of the functional jelly drink, hematological and clinical chemistry parameters were analyzed by the Clinical Laboratory Unit of Srinagarind Hospital, Faculty of Medicine, Khon Kaen University. Any adverse effects, such as nausea or vomiting, abdominal pain, flatulence, heartburn, and diarrhea, were recorded, and their severity was categorized as mild, moderate, or severe.

### 2.15. Statistical Analysis

All results obtained in our study were expressed as mean ± standard deviation (SD). The primary and secondary outcomes were analyzed using intention to treat (ITT) and per-protocol (PP) approaches. The ITT approach was applied to evaluate real-world treatment effectiveness, whereas the PP approach was applied to evaluate biological efficacy under ideal conditions. However, in this study, all participants completed all study visits. Therefore, the ITT and PP analyses were identical. To reduce type I error, a “prioritize pre-planned hypothesis” approach was applied. The hypothesis related to the primary outcome, objective brain wave data obtained from event-related potential (ERP), particularly P300 and N100, was set as the primary hypothesis and was analyzed separately. Other secondary outcomes were analyzed to provide supportive evidence regarding the potential effects of the guava jelly drink. Effect sizes were calculated and presented as Cohen’s d for parametric data and Rosenthal’s r for non-parametric data. Data normality was assessed using the Shapiro–Wilk test. Variables that revealed a normal distribution were analyzed using repeated measures analysis of covariance (ANCOVA), followed by the LSD post hoc test. For gut microbiota, analysis of covariance (ANCOVA) was applied. Non-parametric tests were used to analyze the data that did not follow normal distribution. *p* < 0.05 was used to establish statistical significance. The data analysis was performed using SPSS 26.0.

## 3. Results

### 3.1. Demographic Characteristics of the Participants

In this study, all participants completed the study, and all personal data were analyzed. The demographic characteristics of the participants in all groups are shown in Table 2. No statistically significant differences in age, gender, and vital signs were noted among groups at baseline and throughout the 8-week trial period.

### 3.2. Changes in Cognitive Function

Table 3 reveals that at the Fz location, baseline P300 latency in subjects who consumed the high-dose guava jelly drink was lower than that in the placebo group (*p*-value = 0.048), whereas other parameters did not differ significantly from those in the placebo group. In addition, no significant differences were observed in any parameters at the Cz location. After 1 month of treatment, subjects who consumed either low or high doses of guava jelly drink showed a significant increase in N100 amplitude when compared with the placebo group (*p*-value < 0.05 for all comparisons with the placebo treated group, Cohen’s d = 0.623 and 0.606, respectively). When the consumption period was prolonged to 2 months, these significance changes disappeared. After 2 months of consumption, subjects who consumed low-dose guava jelly drink showed a significant reduction in P300 latency (*p*-value < 0.05 compared with the placebo group, Cohen’s d = 0.792). No other significant changes were observed at the Cz and Fz locations.

In this study, working memory was assessed using a computerized battery test, with the results summarized in Table 4. Prior to product consumption, no significant differences regarding the response time or fluency of any parameters were noted between the groups. After 1 month of consumption, participants who consumed the high-dose guava jelly drink exhibited a significantly faster response time and more fluency in the inhibition test compared with the placebo group (*p*-value < 0.05 compared with the placebo group, Rosenthal’s r = 0.347). When the consumption period was prolonged to 2 months, these significant changes disappeared. However, this group showed significantly improved fluency in the picture recognition test and a shorter response time in the digit vigilance test (*p*-value < 0.05 for all comparisons with the placebo; Rosenthal’s r = 0.320 and Cohen d = 0.40). No other changes were detected.

### 3.3. Changes in Mental Health-Related Parameters

After 2 months of treatment, subjects who consumed high-dose guava jelly drink showed a significantly decreased PSS from baseline (*p*-value = 0.039) (Table 5). No other significant changes were observed.

Table 6 reveals the effect of guava jelly drink on anxiety and depression. After 2 months of treatment, subjects who consumed low-dose guava jelly drink exhibited improved anxiety scores (*p*-value < 0.05; compared to the placebo group, r = 0.444), whereas the high-dose group exhibited improved total HADS scores (*p*-value < 0.05 compared to baseline data, r = 0.395).

### 3.4. Changes in Biochemical Parameters

Table 7 shows that no statistically significant differences in any serum biomarkers associated with neurotransmitter enzymes, including MAO, MAO-A, MAO-B, GABA-T, GAD, and AChE, were observed between the three groups at baseline, 1 month, and 2 months.

The effects of guava jelly drink on serum oxidative stress markers, including MDA, GPx, and CAT, were also explored, and the data are presented in Table 8. No significant differences in these parameters were noted at baseline. In addition, after 1 and 2 months of consumption, all aforementioned biomarkers also failed to produce a significant change from baseline, and no significant differences were noted in all oxidative stress biomarkers used in this study between the groups.

**Figure 2:**
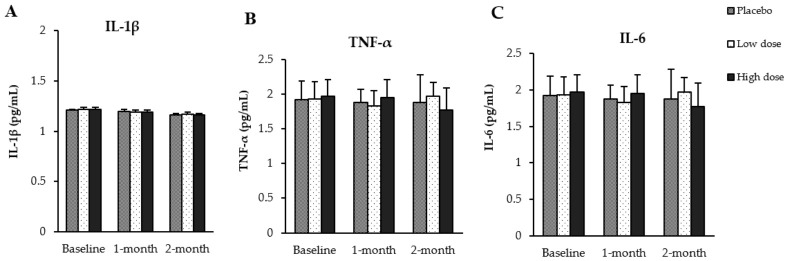
Effects of the guava jelly drinks on inflammatory biomarkers: (**A**) IL-1β, (**B**) TNF-α, and (**C**) IL-6 at baseline, 1 month, and 2 months (placebo, n = 25; low dose, n = 23; high dose, n = 25). Data are presented as mean ± SD.

To explore the possible underlying mechanisms, we also investigate the effects of guava jelly drink on serum inflammatory markers, and results are presented in Figure 2. No significant changes were noted at baseline among the groups, and significant changes in interleukin 1-B, interleukin 6, and TNF-α were noted before the intervention and throughout the 2-month consumption.

The effects of guava jelly drinks on the serum levels of BDNF, cortisol, and total phenolic compounds among groups prior to the intervention and throughout the 2-month consumption period are presented in Table 9. No significant changes in any parameters at baseline were observed among all investigated groups in this study. Furthermore, no significant differences in these parameters were detected throughout the experimental period when compared to placebo group.

### 3.5. Changes in LAB, Lactobacillus spp., and Bifidobacterium spp.

**Figure 3:**
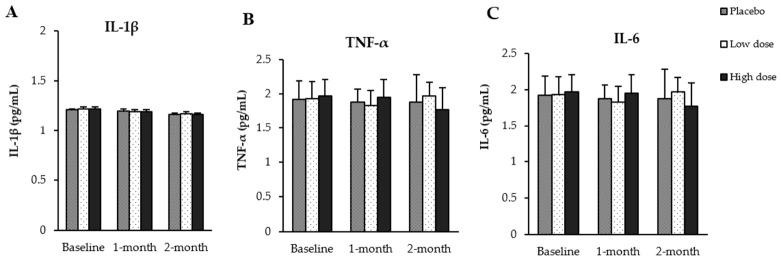
Fecal densities of (**A**) lactic-acid-producing bacteria, (**B**) *Lactobacillus* spp., and (**C**) *Bifidobacterium* spp. in participants who consumed guava jelly drink at baseline and after 2 months of consumption (placebo, n = 25; low dose, n = 23; high dose, n = 25). Data are presented as mean ± SD.

We also explored the effect of guava jelly drink on alterations in probiotic bacteria, including *Lactobacillus* spp. and *Bifidobacterium* spp., as possible modulators of the gut–brain axis [43], and the data are shown in Figure 3. Before the intervention and after 1 and 2 months of consumption, no significant changes in LAB density or the densities of *Lactobacillus* spp. and *Bifidobacterium* spp. were observed between the groups. Compared with baseline, the data also showed no significant changes after 2 months.

### 3.6. Safety Evaluation

To assess the safety of the consumption of the novel guava jelly drink, adverse event and serious side effects were monitored (Table 10). In addition, hematological and clinical chemistry changes are shown in Table 11 and Table 12. Table 10 demonstrates that side effects, including nausea, vomiting, abdominal pain, heartburn, and flatulence, were observed in the placebo group at 1 month. The high-dose guava jelly drink group only experienced nausea, vomiting, flatulence, and heartburn. In contrast, the low-dose group did not report any side effects. When the consumption period was prolonged to 2 months, subjects who consumed placebo and high doses of guava jelly drink experienced only flatulence and heartburn, whereas the low-dose group did not experience any side effects.

Table 11 and Table 12 reveal that subjects who consumed a low dose of the developed drink exhibited significantly higher proportions of eosinophils, whereas the high-dose group exhibited significantly increased total bilirubin levels compared to the placebo group (*p* < 0.05 all comparisons with the placebo group) after 1 month of consumption. Furthermore, participants who consumed the high-dose guava jelly drink for 2 months exhibited significantly decreased mean platelet volume (MPV) (*p*-value < 0.05; compared with the placebo group). No other significant changes in other parameters were observed. All changes observed in this study were still in the normal range.

## 4. Discussion

The current data clearly demonstrate that the consumption of the developed guava jelly drink at low and high doses causes a cognitive-enhancing effect. The low-dose drink improves the N100 amplitude after 1 month of consumption and decreases P300 latency after 2 months of consumption, whereas the high-dose guava jelly drink only improves the N100 amplitude. In addition, high-dose guava jelly drink consumption for 1 month improved response time and fluency in the inhibition test. When consumption was prolonged to 2 months, it improved response time and fluency in the digit vigilance and picture recognition tests, respectively, of the computerized battery test. High-dose guava jelly drink also exhibits an antistress effect by decreasing the PSS after 2 months of consumption. Moreover, guava jelly drink contributed to the positive modulation of mood in healthy subjects. The consumption of low-dose guava jelly drink reveals anxiolytic-liked effect by decreasing the HADS anxiety score, whereas the consumption of a high dose of the developed drink improves the total HADS score. No serious side effects and events were reported throughout the consumption period. Unfortunately, no significant changes in neurotransmitters related to cognition, stress response, and mood regulation; serum levels of oxidative stress and inflammatory markers, such as BDNF and cortisol; and fecal densities of LAB, *Lactobacillus* spp., and *Bifidobacterium* spp. are detected.

An increase in N100 amplitude indicates an improvement in selective attention capability [44], whereas the inhibition test measures inhibitory control or the brain’s ability to stop automatic impulses and resist distractions [45]. Our data reveal that the consumption of high-dose guava jelly drink improves response time and fluency in inhibitory control. This finding indicates that high-dose guava jelly drink improves the brain’s capacity to inhibit automatic impulses and to resist distractors. This subsequently improves selective attention [46], which manifests as an increase in N100 amplitude. Prolonging the intervention period to 2 months improves the reaction time of the digit vigilance test, indicating improvements in alertness, vigilance, and sustained attention [47]. Furthermore, an improvement in fluency in the picture recognition test is also observed after 2 months of consumption of high-dose guava jelly drink, indicating an improvement in the brain’s ability to identify and recognize a visual image [48]. Based on the crucial role of attention in recognition memory [49], we suggest that consumption of high-dose guava jelly drink for 2 months improves recognition memory. In this study, prolonging the consumption of low-dose guava jelly drink to 2 months also improves cognitive processing speed [50]. Beyond the cognitive-enhancing effect, the guava jelly drink also improves psychological well-being [51], particularly antianxiety, which manifests as improvements in the HADS anxiety score and total score.

Factors that play crucial roles in cognition, working memory, stress response, and mood regulation, including oxidative stress [52,53,54,55,56], inflammation [57,58,59,60], main classical neurotransmitters [61,62,63,64], brain-derived neurotrophic factor (BDNF) [65,66,67], stress hormones and cortisol [64,67,68,69], and gut microbiota [70,71,72,73], were also investigated in this study to explore the possible underlying mechanisms. However, our current data did not show significant changes in the aforementioned factors. A possible explanation for this lack of significant findings may involve study limitations. In this study, oxidative stress, inflammation, and classical neurotransmitters were assessed in the peripheral system, specifically in serum. Serum levels of these biomarkers have previously been reported to be associated with their levels in the brain [74,75,76]. Because direct assessment in the brain or in cerebrospinal fluid is invasive and is not appropriate for healthy individuals without clinical indications, we measured these parameters in the serum. However, the levels of these biomarkers may not only reflect oxidative stress, inflammation, and classical neurotransmitter changes in the brain. They may also reflect contributions from other tissues, particularly muscle, which represents the largest tissue mass in the human body. Therefore, peripheral biomarkers have limited utility for inferring brain oxidative stress status, inflammation, and neurotransmitter activity. In addition, neurotransmitters in this study were assessed using an indirect method that measured the activities of enzymes that play a pivotal role in the inactivation of classical neurotransmitters. Given these limitations, direct, non-invasive brain imaging of these parameters should be performed using positron-emission tomography (PET). PET can be used to measure oxidative stress status, particularly superoxide radical (O^-^_2_), a major oxidative stress species in the body that is produced by mitochondria, using special tracers such as [^18^F]ROStrace [77]. PET can also assess inflammation using translocator protein (TSPO) tracers, such as ^11^C-PBR28 [78], ^11^C-DPA713 [79], and DPA-714 [80]. Moreover, PET can also measure various types of neurotransmitters using specific tracers [81]. Thus, PET provides information regarding the effect of guava jelly drink on parameters such as oxidative stress, inflammation, and various neurotransmitters, thereby providing more precise insight into the underlying mechanisms of the guava jelly drink. However, PET studies are very expensive and difficult to perform. Therefore, further exploration is required.

For microbiota assessment, we measured only lactic acid-producing bacteria (LAB) such as *Lactobacillus* species and *Bifidobacterium* species. Therefore, the obtained data did not reflect changes in all microbiota species, such as *Akkermansia*, that are involved in improving cognition, working memory, stress, and anxiety [82,83,84]. Future studies should assess other species, such as *Akkermansia*, that potentially play a role in these processes. Moreover, other studies also demonstrated that neuropeptides, such as oxytocin, vasopressin, neuropeptide Y and S, and substance P, affect components of mood, such as anxiety and depression [85], together with memory [86] and stress responses [87]. Therefore, we suggest that the guava jelly drink potentially exhibits cognitive-enhancing, antistress, and anxiolytic-liked effects by modulating neuropeptide concentrations. However, this hypothesis requires further exploration.

Accumulating evidence has demonstrated that polyphenolic compounds, flavonoids, and vitamin C improve cognition, working memory, stress, and anxiety [82,83,84,88,89,90]. Our developed functional drink is rich in these substances; however, their concentrations appear to be lower than the doses that were previously reported to produce cognitive-enhancing, antistress, and antianxiety effects [82,83,84,88,89,90]. Therefore, it is less likely that the positive modulation effects of the developed guava jelly drink observed in this study occur as a result of a single ingredient. We suggest that the interactions between various ingredients in the product may account for the positive modulation effects of the developed drink. However, a precise understanding of the potential active ingredients is also required to address this issue.

The current results do not show a dose-dependent effect. A possible explanation is that a linear relationship may not exist between the concentration of the functional ingredients (guava juice and honey mint syrup) used in this study and the observed parameters. All measured parameters in this study are under the influence of many factors, including the concentrations of the functional ingredients. Furthermore, the guava juice and mint syrup contain various ingredients, potentially including active ingredients and nonactive-ingredients. Thus, the increasing concentrations of the tested product can mask the effects of the potential active ingredients.

Given that the developed guava jelly drink is categorized as novel food, safety evaluation is essential. Because the changes in all of the observed parameters were within a normal range, no toxicity was noted in association with this novel drink. In addition, no adverse effect and serious side effects were detected. Therefore, this novel drink is safe for consumption.

A strength of this study is the well-controlled management of confounding factors, such as physical activity [91] and the types of food consumed [92] by recording both factors (Supplementary Material Files S1 and S2). Based on this information, it can be assured that the positive modulation effects of guava jelly drink observed in this study are the result of the guava jelly drink intervention. In addition, randomization, blinding, and concealment were also well-controlled. However, a larger sample size is also required to confirm the results, and a long-term intervention study is also needed to establish safety following long-term application and the sustained effectiveness of consumption.

## 5. Conclusions

The current study clearly demonstrates that the novel guava jelly drink is safe for consumption. Consumption of the guava jelly drink at a high dose (73.2%) for 8 weeks improves inhibitory control and the brain’s capacity to inhibit automatic impulses and resist distractors. This subsequently improves selective attention by enhancing cognition, particularly in the working memory domain. Moreover, the low-dose (36.6%) drink improves cognitive function by increasing cognitive processing speed. In addition, the high dose of this novel functional drink also exhibits antistress and antianxiety effects. The precise mechanism still requires further exploration.

## Figures and Tables

**Table 1:** The ingredients of placebo, high-dose, and low-dose guava jelly drink.

Ingredients	Placebo (%)	High Dose (%)	Low Dose (%)
Guava juice	0.00	63.55	31.78
Mint syrup	0.00	9.68	4.84
Honey syrup	9.68	0.00	4.84
PPDF	0.14	0.14	0.14
Agar	0.54	0.54	0.54
Ascorbic acid	0.05	0.05	0.05
Pear puree	5.45	5.45	5.45
Water	84.13	20.58	52.36
Total	100.0	100.0	100.0

**Table 2:** Demographic characteristics of participants at baseline, 1 month, and 2 months of treatment. Data are reported as mean ± SD.

Parameters	Time	Placebo (N = 25)	Low Dose (N = 23)	High Dose (N = 25)
Age (year)	Baseline	31.04 ± 5.96	28.35 ± 6.13 (*p* = 0.121)	29.20 ± 6.65 (*p* = 0.280)
Gender (Male/Female)	Baseline	9/16	10/13	10/15
Blood temperature (°C)	Baseline	36.62 ± 0.12	36.56 ± 0.25 (*p* = 0.472)	36.55 ± 0.33 (*p* = 0.831)
	1-month	36.60 ± 0.13	36.57 ± 0.15 (*p* = 0.121)	36.54 ± 0.27 (*p* = 0.629)
	2-month	36.58 ± 0.20	36.59 ± 0.19 (*p* = 0.659)	36.63 ± 0.17 (*p* = 0.306)
Heart rate (beats/min)	Baseline	77.44 ± 11.72	74.96 ± 8.97 (*p* = 0.440)	74.04 ± 12.10 (*p* = 0.281)
	1-month	73.84 ± 9.87	76.09 ± 10.17 (*p* = 0.439)	76.88 ± 9.92 (*p* = 0.285)
	2-month	75.16 ± 9.87	76.13 ± 8.50 (*p* = 0.744)	75.68 ± 11.89 (*p* = 0.858)
Respiratory rate (breaths/min)	Baseline	17.40 ± 0.50	17.35 ± 0.57 (*p* = 0.810)	17.16 ± 0.47 (*p* = 0.094)
	1-month	17.52 ± 0.59	17.48 ± 0.59 (*p* = 0.409)	17.56 ± 0.51 (*p* = 0.902)
	2-month	17.32 ± 0.80	17.26 ± 0.62 (*p* = 0.589)	17.36 ± 0.64 (*p* = 0.966)
Systolic BP (mmHg)	Baseline	112.76 ± 10.24	117.04 ± 8.13 (*p* = 0.148)	113.64 ± 11.61 (*p* = 0.760)
	1-month	110.56 ± 11.21	115.13 ± 8.04 (*p* = 0.796)	114.52 ± 9.34 (*p* = 0.260)
	2-month	110.24 ± 11.38	115.04 ± 6.15 (*p* = 0.077)	110.60 ± 9.31(*p* = 0.891)
Diastolic BP (mmHg)	Baseline	72.52 ± 7.46	73.22 ± 7.16 (*p* = 0.754)	73.12 ± 8.33 (*p* = 0.783)
	1-month	72.04 ± 8.33	73.26 ± 7.04 (*p* = 0.603)	72.88 ± 8.70 (*p* = 0.714)
	2-month	70.60 ± 8.66	72.26 ± 9.87 (*p* = 0.510)	71.48 ± 7.42 (*p* = 0.721)

**Table 3:** The latency and amplitude values of the N100 and P300 waves derived from event-related potentials (ERPs) in volunteers who consumed functional jelly drinks at baseline, 1-month, and 2-month period.

Location	Wave	Group	Baseline	1 Month	2-Month
**Fz**	**N100 Latency (ms)**	Placebo (N = 25)	112.04 ± 4.38	109.80 ± 4.92	110.40 ± 5.48
		Low dose (N = 23)	110.09 ± 6.30 (*p* = 0.263)	109.87 ± 5.12 (*p* = 0.942)	109.83 ± 6.24 (*p* = 0.756)
		High dose (N = 25)	109.76 ± 5.28 (*p* = 0.136)	108.40 ± 4.73 (*p* = 0.315)	111.16 ± 5.20 (*p* = 0.647)
	**N100 Amplitude (μV)**	Placebo (N = 25)	4.45 ± 1.64	4.77 ± 1.96	5.22 ± 2.04
		Low dose (N = 23)	4.05 ± 1.74 (*p* = 0.397)	5.32 ± 1.98 (*p* = 0.348)	5.07 ± 1.92 (*p* = 0.807)
		High dose (N = 25)	4.53 ± 2.10 (*p* = 0.749)	4.36 ± 1.75 (*p* = 0.541)	5.87 ± 2.13 (*p* = 0.262)
	**P300 Latency (ms)**	Placebo (N = 25)	339.16 ± 12.12	−0.50 ± 0.71 ^a^	1.05 ± 0.82 ^a^
		Low dose (N = 23)	333.78 ±9.21(*p* = 0.106)	1.00 ± 0.82 ^a^ (*p* = 0.191)	0.74 ± 0.78 ^a^ (*p* = 0.773)
		High dose (N = 25)	332.68 ± 12.37 * (*p* = 0.048)	1.72 ± 0.86 ^a^ (*p* = 0.050)	1.80 ± 0.65 ^a^ (*p* = 0.476)
	**P300 Amplitude (μV)**	Placebo (N = 25)	30.57 ± 4.51	28.46 ± 3.41	31.00 ± 4.73
		Low dose (N = 23)	29.78 ± 5.12 (*p* = 0.613)	29.79 ± 4.72 (*p* = 0.307)	31.42 ± 5.28 (*p* = 0.796)
		High dose (N = 25)	28.59 ± 6.11 (*p* = 0.357)	28.83 ± 5.12 (*p* = 0.768)	31.98 ± 3.64 (*p* = 0.635)
**Cz**	**N100 Latency (ms)**	Placebo (N = 25)	109.64 ± 6.10	109.24 ± 5.66	109.96 ± 5.31
		Low dose (N = 23)	110.26 ± 4.86 (*p* = 0.724)	108.09 ± 5.33 (*p* = 0.344)	107.48 ± 6.02 (*p* = 0.118)
		High dose (N = 25)	108.88 ± 6.95 (*p* = 0.659)	108.00 ± 5.68 (*p* = 0.430)	107.84 ± 4.95 (*p* = 0.172)
	**N100 Amplitude (μV)**	Placebo (N = 25)	4.43 ± 1.64	4.45 ± 1.46	5.20 ± 2.00
		Low dose (N = 23)	4.75 ± 1.76 (*p* = 0.380)	5.53 ± 1.99 * (*p* = 0.048; d = 0.623)	5.25 ± 2.25 (*p* = 0.885)
		High dose (N = 25)	4.89 ± 1.93 (*p* = 0.299)	5.55 ± 2.11 * (*p* = 0.041; d = 0.606)	5.44 ± 2.25 (*p* = 0.698)
	**P300 Latency (ms)**	Placebo (N = 25)	337.60 ± 13.00	340.04 ± 9.09	343.92 ± 8.77
		Low dose (N = 23)	337.13 ± 10.78 (*p* = 0.679)	340.70 ± 12.14 (*p* = 0.749)	334.96 ± 13.56 * (*p* = 0.011; d = 0.792)
		High dose (N = 25)	337.80 ± 11.09 (*p* = 0.838)	338.00 ± 9.81 (*p* = 0.641)	337.28 ± 12.75 (*p* = 0.051)
	**P300 Amplitude (μV)**	Placebo (N = 25)	26.80 ± 5.87	27.04 ± 6.00	28.07 ± 6.27
		Low dose (N = 23)	27.61 ± 5.05 (*p* = 0.626)	29.04 ± 5.87 (*p* = 0.183)	29.91 ± 6.53 (*p* = 0.304)
		High dose (N = 25)	27.19 ± 6.00 (*p* = 0.808)	25.91 ± 5.63 (*p* = 0.503)	28.41 ± 5.60 (*p* = 0.843)

Data are reported as mean ± SD. * *p* < 0.05 compared with placebo. d = Cohen’s d effect size. ^a^ The results were normalized and presented as percent change from baseline values.

**Table 4:** Effect of various doses of guava jelly drink on working memory as assessed using the computerized battery test (CDR) at baseline 1 month and 2 months.

Cognitive Domains	Test Items	Group	Baseline	1-Month	2-Month
**Simple** **reaction time**	Response time	Placebo (N = 25)	0.018 ± 0.01	0.016 ± 0.00	0.018 ± 0.01
		Low dose (N = 23)	0.02 ± 0.01 (*p* = 0.427)	0.017 ± 0.01 (*p* = 0.877)	0.015 ± 0.00 (*p* = 0.556)
		High dose (N = 25)	0.019 ± 0.02 (*p* = 0.042)	0.016 ± 0.01 (*p* = 0.567)	0.017 ± 0.01 (*p* = 0.771)
	Fluency	Placebo (N = 25)	8.98 ± 1.96	8.5 ± 2.39	8.88 ± 1.92
		Low dose (N = 23)	9.27 ± 0.58 (*p* = 0.613)	9.17 ± 1.14 (*p* = 0.261)	9.20 ± 0.59 (*p* = 0.877)
		High dose (N = 25)	9.31 ± 0.86 (*p* = 0.662)	9.41 ± 0.62 (*p* = 0.118)	8.70 ± 2.08 (*p* = 0.884)
**Inhibition**	Response time	Placebo (N = 25)	0.016 ± 0.01	0.021 ± 0.01	0.020 ± 0.01
		Low dose (N = 23)	0.016 ± 0 (*p* = 0.613)	0.018 ± 0.01 (*p* = 0.984)	0.018 ± 0.01 (*p* = 0.642)
		High dose (N = 25)	0.015 ± 0.01 (*p* = 0.503)	0.016 ± 0.01 (*p* = 0.157)	0.019 ± 0.01 (*p* = 0.497)
	Fluency	Placebo (N = 25)	10.13 ± 0.6	8.57 ± 3.37	8.01 ± 3.58
		Low dose (N = 23)	10.27 ± 0.36 (*p* = 0.781)	9.73 ± 1.82 (*p* = 0.055)	9.84 ± 1.52 (*p* = 0.146)
		High dose (N = 25)	10.03 ± 1.12 (*p* = 0.541)	10.01 ± 0.91 * (*p* = 0.014; r = 0.347)	10.08 ± 0.62 (*p* = 0.062)
**Word** **recognition**	Response time	Placebo (N = 25)	0.031 ± 0.01	0.030 ± 0.01	0.028 ± 0.01
		Low dose (N = 23)	0.033 ± 0.01 (*p* = 0.62)	0.028 ± 0.01 (*p* = 0.516)	0.028 ± 0.01 (*p* = 0.765)
		High dose (N = 25)	0.030 ± 0.01 (*p* = 0.607)	0.031 ± 0.01 (*p* = 0.892)	0.029 ± 0.01 (*p* = 0.72)
	Fluency	Placebo (N = 25)	16.04 ± 2.72	16.45 ± 2.32	16.97 ± 2.03
		Low dose (N = 23)	16.29 ± 1.86 (*p* = 0.765)	16.70 ± 2.28 (*p* = 0.796)	16.83 ± 1.7 (*p* = 0.992)
		High dose (N = 25)	15.23 ± 3.22 (*p* = 0.554)	16.59 ± 2.42 (*p* = 0.594)	16.12 ± 3.88 (*p* = 0.778)
**Picture** **recognition**	Response time	Placebo (N = 25)	0.027 ± 0.01	0.024 ± 0.01	0.026 ± 0.01
		Low dose (N = 23)	0.026 ± 0.01 (*p* = 0.65)	0.026 ± 0.01 (*p* = 0.516)	0.026 ± 0.00 (*p* = 0.556)
		High dose (N = 25)	0.026 ± 0.01 (*p* = 0.607)	0.026 ± 0.01 (*p* = 0.225)	0.024 ± 0.01 (*p* = 0.177)
	Fluency	Placebo (N = 25)	17.82 ± 1.96	18.31 ± 1.17	17.47 ± 2.38
		Low dose (N = 23)	17.89 ± 1.21 (*p* = 0.812)	17.98 ± 0.92 (*p* = 0.439)	18.46 ± 1.03 (*p* = 0.157)
		High dose (N = 25)	17.91 ± 1.15 (*p* = 0.648)	18.29 ± 1.18 (*p* = 0.607)	18.6 ± 1.12 * (*p* = 0.024; r = 0.320)
**Digit** **vigilance**	Response time	Placebo (N = 25)	0.018 ± 0.01	0.018 ± 0.01	0.021 ± 0.01
		Low dose (N = 23)	0.019 ± 0.01 (*p* = 0.588)	0.017 ± 0.01 (*p* = 0.906)	0.021 ± 0.01 (*p* = 0.734)
		High dose (N = 25)	0.018 ± 0.01 (*p* = 0.918)	0.019 ± 0.01 (*p* = 0.653)	0.017 ± 0.01 * (*p* = 0.048; d = 0.400)
	Fluency	Placebo (N = 25)	10.36 ± 0.62	10.01 ± 2.15	10.27 ± 1.25
		Low dose (N = 23)	9.80 ± 1.79 (*p* = 0.094)	11.63 ± 6.99 (*p* = 0.464)	12.15 ± 5.9 (*p* = 0.657)
		High dose (N = 25)	12.87 ± 11.76 (*p* = 0.497)	10.15 ± 0.8 (*p* = 0.567)	10.38 ± 0.82 (*p* = 0.522)
**Spatial working memory**	Response time	Placebo (N = 25)	0.025 ± 0.01	0.024 ± 0.01	0.025 ± 0.00
		Low dose (N = 23)	0.026 ± 0.01 (*p* = 0.248)	0.023 ± 0.01 (*p* = 0.332)	0.025 ± 0.01 (*p* = 0.893)
		High dose (N = 25)	0.025 ± 0.01 (*p* = 0.923)	0.024 ± 0.01 (*p* = 0.992)	0.025 ± 0.01 (*p* = 0.969)
	Fluency	Placebo (N = 25)	23.31 ± 5.81	23.28 ± 6.06	26.27 ± 4.35
		Low dose (N = 23)	19.62 ± 7.75 (*p* = 0.114)	23.52 ± 6.84 (*p* = 0.476)	25.65 ± 6.61 (*p* = 0.703)
		High dose (N = 25)	22.85 ± 6.19 (*p* = 0.9)	24.46 ± 5.87 (*p* = 0.421)	25.03 ± 5.29 (*p* = 0.455)
**Choice** **reaction time**	Response time	Placebo (N = 25)	0.022 ± 0.01	0.021 ± 0.01	0.021 ± 0.01
		Low dose (N = 23)	0.021 ± 0.01 (*p* = 0.926)	0.020 ± 0.01 (*p* = 0.812)	0.022 ± 0.01 (*p* = 0.364)
		High dose (N = 25)	0.022 ± 0.01 (*p* = 0.641)	0.020 ± 0.01 (*p* = 0.771)	0.021 ± 0.01 (*p* = 0.684)
	Fluency	Placebo (N = 25)	15.37 ± 3.76	16.96 ± 2.87	16.41 ± 2.29
		Low dose (N = 23)	15.68 ± 3.43 (*p* = 0.464)	16.36 ± 3.03 (*p* = 0.337)	15.85 ± 2.88 (*p* = 0.672)
		High dose (N = 25)	16.25 ± 3.07 (*p* = 0.528)	16.23 ± 2.89 (*p* = 0.09)	16.59 ± 2.65 (*p* = 0.404)

Data are reported as mean ± SD. * *p* < 0.05 compared with placebo. d: Cohen’s d effect size; r: Rosenthal’s r effect size.

**Table 5:** Changes in Perceived Stress Scale (PSS) scores from baseline after 1 and 2 months of treatment in participants consuming functional jelly drinks.

Perceived Stress Scale (PSS)	Placebo (N = 25)	Low Dose (N = 23)	High Dose (N = 25)
Baseline	18.20 ± 5.75	19.91 ± 3.94 (*p* = 0.191)	19.92 ± 3.39 (*p* = 0.180)
% change from baseline to 1 month	14.11 ± 10.97	−2.71 ± 4.13 (*p* = 0.111)	−5.86 ± 3.46 (*p* = 0.051)
% change from baseline to 2 months	12.41 ± 9.93	−8.30 ± 5.71 (*p* = 0.274)	−11.03 ± 5.92 * (*p* = 0.039; r = 0.292)

Data are reported as mean ± SD. * *p* < 0.05 compared with baseline. r: Rosenthal’s r effect size.

**Table 6:** Hospital Anxiety and Depression Scale (HADS) scores of participants consuming various doses of guava jelly drinks at baseline, 1-month and 2-month consumption period.

HADS	Group	Baseline	1-Month	2-Month
Anxiety	Placebo (N = 25)	4.56 ± 1.69	4.28 ± 2.37 (*p* = 0.414)	3.88 ± 2.24 (*p* = 0.121)
	Low dose (N = 23)	5.00 ± 1.48	4.22 ± 2.41 (*p* = 0.184)	3.87 ± 2.69 * (*p* = 0.033; r = 0.444)
	High dose (N = 25)	4.52 ± 2.14	4.12 ± 2.07 (*p* = 0.348)	3.36 ± 2.48 (*p* = 0.052)
Depression	Placebo (N = 25)	3.40 ± 1.50	2.84 ± 2.21 (*p* = 0.148)	2.92 ± 2.04 (*p* = 0.196)
	Low dose (N = 23)	3.61 ± 1.37	3.96 ± 2.84 (*p* = 0.894)	3.48 ± 2.25 (*p* = 0.725)
	High dose (N = 25)	3.48 ± 1.39	3.00 ± 1.55 (*p* = 0.185)	2.96 ± 1.93 (*p* = 0.306)
Total	Placebo (N = 25)	7.96 ± 2.54	7.12 ± 3.93 (*p* = 0.116)	6.80 ± 3.84 (*p* = 0.105)
	Low dose (N = 23)	8.61 ± 1.97	8.17 ± 4.63 (*p* = 0.174)	7.35 ± 4.29 (*p* = 0.152)
	High dose (N = 25)	8.00 ± 2.48	7.12 ± 3.33 (*p* = 0.163)	6.32 ± 3.76 * (*p* = 0.048; r = 0.395)

Data are reported as mean ± SD. * *p* < 0.05 compared with baseline. r: Rosenthal’s r effect size.

**Table 7:** Effects of guava jelly drinks on serum biomarkers related to neurotransmitter enzymes in participants at baseline, 1 month and 2 months. Data are reported as mean ± SD.

Parameters	Group	Baseline	1-Month	2-Month
MAO (μmol/mg protein)	Placebo (N = 25)	0.95 ± 0.47	1.01 ± 0.47	1.11 ± 0.69
	Low dose (N = 23)	0.91 ± 0.50 (*p* = 0.848)	0.93 ± 0.41 (*p* = 0.388)	0.94 ± 0.43 (*p* = 0.551)
	High dose (N = 25)	0.97 ± 0.45 (*p* = 0.695)	0.98 ± 0.41 (*p* = 0.826)	1.04 ± 0.47 (*p* = 0.837)
MAO-A (μmol/mg protein)	Placebo (N = 25)	0.82 ± 0.43	0.82 ± 0.38	0.93 ± 0.52
	Low dose (N = 23)	0.81 ± 0.49 (*p* = 0.733)	0.75 ± 0.28 (*p* = 0.46)	0.82 ± 0.31 (*p* = 0.566)
	High dose (N = 25)	0.77 ± 0.37 (*p* = 1)	0.76 ± 0.31 (*p* = 0.551)	0.82 ± 0.33 (*p* = 0.711)
MAO-B (μmol/mg protein)	Placebo (N = 25)	0.84 ± 0.43	0.7 ± 0.33	0.89 ± 0.51
	Low dose (N = 23)	0.86 ± 0.50 (*p* = 0.983)	0.66 ± 0.28 (*p* = 0.555)	0.77 ± 0.31 (*p* = 0.61)
	High dose (N = 25)	0.81 ± 0.34 (*p* = 0.757)	0.66 ± 0.29 (*p* = 0.676)	0.77 ± 0.34 (*p* = 0.621)
GABA-T (nmol/mg protein)	Placebo (N = 25)	0.12 ± 0.06	0.12 ± 0.06	0.12 ± 0.08
	Low dose (N = 23)	0.12 ± 0.07 (*p* = 0.882)	0.11 ± 0.05 (*p* = 0.633)	0.11 ± 0.05 (*p* = 0.915)
	High dose (N = 25)	0.11 ± 0.05 (*p* = 0.885)	0.11 ± 0.05 (*p* = 0.582)	0.11 ± 0.05 (*p* = 0.869)
GAD (ng/mL)	Placebo (N = 25)	0.38 ± 0.09	3.01 ± 0.51	3.13 ± 0.58
	Low dose (N = 23)	0.40 ± 0.15 (*p* = 0.861)	3.15 ± 0.88 (*p* = 0.930)	3.34 ± 0.69 (*p* = 0.359)
	High dose (N = 25)	0.38 ± 0.69 (*p* = 0.828)	3.04 ± 0.42 (*p* = 0.782)	2.89 ± 0.60 (*p* = 0.297)
AChE (nmol/mg protein)	Placebo (N = 25)	0.08 ± 0.05	0.08 ± 0.04	0.09 ± 0.06
	Low dose (N = 23)	0.08 ± 0.05 (*p* = 0.718)	0.08 ± 0.03 (*p* = 0.482)	0.08 ± 0.03 (*p* = 0.949)
	High dose (N = 25)	0.08 ± 0.03 (*p* = 0.853)	0.08 ± 0.03 (*p* = 0.509)	0.08 ± 0.04 (*p* = 0.789)

**Table 8:** Effects of guava jelly drink on oxidative stress biomarkers at baseline, 1 month, and 2 months.

Parameters	Group	Baseline	1-Month	2-Month
MDA (μmol/mg protein)	Placebo (N = 25)	0.48 ± 0.14	0.37 ± 0.09	0.38 ± 0.07
	Low dose (N = 23)	0.55 ± 0.16 (*p* = 0.359)	0.40 ± 0.11 (*p* = 0.449)	0.40 ± 0.09 (*p* = 0.593)
	High dose (N = 25)	0.48 ± 0.18 (*p* = 0.252)	0.35 ± 0.09 (*p* = 0.411)	0.36 ± 0.09 (*p* = 0.395)
GPx (U/mg protein)	Placebo (N = 25)	0.10 ± 0.02	0.15 ± 0.06	0.24 ± 0.07
	Low dose (N = 23)	0.10 ± 0.01 (*p* = 0.432)	0.20 ± 0.08 (*p* = 0.098)	0.24 ± 0.05 (*p* = 0.962)
	High dose (N = 25)	0.09 ± 0.01 (*p* = 0.937)	0.21 ± 0.08 * (*p* = 0.020)	0.23 ± 0.07 (*p* = 0.687)
CAT (U/mg protein)	Placebo (N = 25)	1.04 ± 0.57	0.95 ± 0.35	1.15 ± 0.20
	Low dose (N = 23)	0.89 ± 0.71 (*p* = 0.206)	0.93 ± 0.47 (*p* = 0.906)	1.33 ± 0.29 (*p* = 0.071)
	High dose (N = 25)	0.93 ± 0.49 (*p* = 0.607)	0.82 ± 0.36 (*p* = 0.373)	1.07 ± 0.30 (*p* = 0.394)

Data are presented as mean ± SD. * *p* < 0.05 compared with the placebo group.

**Table 9:** Effects of guava jelly drinks on serum levels of BDNF, cortisol, and total phenolic compounds in participants at baseline, 1 month, and 2 months.

Parameters	Group	Baseline	1-Month	2-Month
BDNF (ng/mL)	Placebo (N = 25)	61.53 ± 15.27	57.67 ± 15.81	63.99 ± 22.57
	Low dose (N = 23)	75.01 ± 26.43 (*p* = 0.183)	65.09 ± 25.49 (*p* = 0.304)	67.01 ± 17.65 (*p* = 0.724)
	High dose (N = 25)	67.61 ± 22.18 (*p* = 0.580)	63.43 ± 15.25 (*p* = 0.408)	66.83 ± 36.64 (*p* = 0.637)
Cortisol (μg/dL)	Placebo (N = 25)	10.90 ± 7.66	11.23 ± 6.89	10.96 ± 6.43
	Low dose (N = 23)	11.23 ± 5.58 (*p* = 0.252)	12.53 ± 7.22 (*p* = 0.358)	11.91 ± 6.67 (*p* = 0.655)
	High dose (N = 25)	9.20 ± 3.76 (*p* = 0.977)	9.43 ± 4.02 (*p* = 0.607)	9.72 ± 5.00 (*p* = 0.542)
Total phenolic compounds (mg Gallic acid/mL)	Placebo (N = 25)	0.74 ± 0.08	0.76 ± 0.06	0.47 ± 0.03
	Low dose (N = 23)	0.72 ± 0.06 (*p* = 0.468)	0.77 ± 0.06 (*p* = 0.733)	0.47 ± 0.03 (*p* = 0.805)
	High dose (N = 25)	0.72 ± 0.06 (*p* = 0.353)	0.73 ± 0.05 (*p* = 0.087)	0.47 ± 0.04 (*p* = 0.717)

Data are reported as mean ± SD.

**Table 10:** Adverse effects in participants who consumed functional jelly drinks for 1 and 2 months. Data are reported as frequency (percentage).

Adverse Effects	1-Month			2-Month		
	Placebo (N = 25)	Low Dose (N = 23)	High Dose (N = 25)	Placebo (N = 25)	Low Dose (N = 23)	High Dose (N = 25)
Nausea, vomiting	1 (4%) Moderate	0	2 (8%) Mild	0	0	0
Abdominal pain	1 (4%) Moderate	0	0	0	0	0
Flatulence	2 (8%) Mild (1) Moderate (1)	0	1 (4%) Mild	1 (4%) Mild	0	1 (4%) Moderate
Heartburn	1 (4%) Moderate	0	2 (8%) Mild	1 (4%) Mild	0	1 (4%) Moderate
Diarrhea	2 (8%) Moderate	0	0	0	0	0

**Table 11:** Hematological changes in participants consuming functional jelly drinks at baseline, 1 month, and 2 months.

Parameters	Reference	Time	Placebo (N = 25)	Low Dose (N = 23)	High Dose (N = 25)
Hemoglobin	12.0–16.7 g/dL	Baseline	12.84 ± 0.91	13.66 ± 1.32 (*p* = 0.077)	13.49 ± 1.50 (*p* = 0.176)
		1-month	13.43 ± 0.80	13.18 ± 0.72 (*p* = 0.264)	13.11 ± 0.70 (*p* = 0.151)
		2-month	13.45 ± 0.90	13.15 ± 0.86 (*p* = 0.245)	13.15 ± 0.85 (*p* = 0.228)
Hematocrit	40.5–50.8%	Baseline	40.95 ± 3.03	43.10 ± 3.87 (*p* = 0.117)	43.05 ± 4.03 (*p* = 0.120)
		1-month	42.25 ± 3.85	42.23 ± 3.60 (*p* = 0.981)	41.87 ± 3.60 (*p* = 0.724)
		2-month	42.91 ± 4.05	42.81 ± 3.84 (*p* = 0.930)	42.54 ± 3.80 (*p* = 0.742)
White blood cell	4.6–10.6 10^3^/μL	Baseline	6.96 ± 0.97	7.40 ± 1.92 (*p* = 0.663)	6.80 ± 1.62 (*p* = 0.447)
		1-month	7.16 ± 1.16	7.14 ± 1.58 (*p* = 0.975)	6.83 ± 1.31 (*p* = 0.397)
		2-month	6.83 ± 1.52	6.93 ± 1.60 (*p* = 0.831)	6.86 ± 1.44 (*p* = 0.535)
Platelets	173–383 10^3^/μL	Baseline	296.46 ± 65.51	300.13 ± 60.14 (*p* = 0.702)	269.88 ± 45.99 (*p* = 0.194)
		1-month	278.16 ± 52.77	286.35 ± 61.10 (*p* = 0.611)	274.96 ± 52.62 (*p* = 0.839)
		2-month	294.25 ± 70.71	296.78 ± 67.74 (*p* = 0.894)	277.04 ± 56.20 (*p* = 0.358)
MPV	8.7–12.5 fL	Baseline	10.57 ± 0.63	10.40 ± 0.87 (*p* = 0.465)	10.26 ± 0.89 (*p* = 0.177)
		1-month	10.53 ± 0.65	10.45 ± 1.00 (*p* = 0.769)	10.29 ± 0.81 (*p* = 0.322)
		2-month	10.62 ± 0.75	10.27 ± 0.99 (*p* = 0.175)	10.10 ± 0.82 * (*p* = 0.040)
Red Blood Cell	4.7–6.2 10^6^/μL	Baseline	4.85 ± 0.89	5.20 ± 0.74 (*p* = 0.242)	5.02 ± 0.52 (*p* = 0.447)
		1-month	4.88 ± 0.88	5.14 ± 0.69 (*p* = 0.269)	4.92 ± 0.60 (*p* = 0.892)
		2-month	5.04 ± 0.73	5.15 ± 0.81 (*p* = 0.610)	4.93 ± 0.53 (*p* = 0.795)
MCV	80.0–97.8 fL	Baseline	82.70 ± 8.06	84.16 ± 10.83 (*p* = 0.584)	86.17 ± 8.19 (*p* = 0.186)
		1-month	83.18 ± 7.06	82.12 ± 14.42 (*p* = 0.728)	86.58 ± 8.90 (*p* = 0.254)
		2-month	84.50 ± 8.03	85.13 ± 11.15 (*p* = 0.444)	87.50 ± 8.58 (*p* = 0.136)
MCH	25.2–32.0 pg	Baseline	25.57 ± 2.97	26.38 ± 3.23 (*p* = 0.353)	26.96 ± 2.72 (*p* = 0.105)
		1-month	25.94 ± 2.76	26.59 ± 3.29 (*p* = 0.447)	27.19 ± 2.73 (*p* = 0.136)
		2-month	26.01 ± 2.89	26.48 ± 3.35 (*p* = 0.590)	27.13 ± 2.68 (*p* = 0.194)
MCHC	31.3–33.4 g/dL	Baseline	30.86 ± 1.02	31.41 ± 0.93 (*p* = 0.092)	31.30 ± 0.95 (*p* = 0.246)
		1-month	31.16 ± 0.96	31.58 ± 0.84 (*p* = 0.122)	31.44 ± 0.97 (*p* = 0.289)
		2-month	30.74 ± 0.98	31.15 ± 1.07 (*p* = 0.185)	31.02 ± 1.09 (*p* = 0.357)
RDW	11.9–14.8%	Baseline	14.50 ± 1.58	13.78 ± 2.08 (*p* = 0.153)	13.60 ± 1.44 (*p* = 0.07)
		1-month	14.58 ± 1.79	13.96 ± 2.19 (*p* = 0.092)	13.75 ± 1.35 (*p* = 0.101)
		2-month	14.54 ± 1.51	13.90 ± 2.15 (*p* = 0.058)	13.94 ± 1.62 (*p* = 0.112)
% Neutrophil	43.7–70.9%	Baseline	54.59 ± 8.00	55.32 ± 8.82 (*p* = 0.781)	52.84 ± 9.92 (*p* = 0.497)
		1-month	56.39 ± 7.82	54.30 ± 6.64 (*p* = 0.303)	52.82 ± 6.42 (*p* = 0.075)
		2-month	55.07 ± 8.77	54.70 ± 6.51 (*p* = 0.868)	53.45 ± 6.84 (*p* = 0.451)
% Lymphocytes	20.1–44.5%	Baseline	36.12 ± 6.81	34.03 ± 8.40 (*p* = 0.325)	34.88 ± 6.29 (*p* = 0.550)
		1-month	34.15 ± 6.47	34.78 ± 6.20 (*p* = 0.717)	36.16 ± 5.26 (*p* = 0.240)
		2-month	35.37 ± 8.68	34.62 ± 5.84 (*p* = 0.716)	35.32 ± 6.35 (*p* = 0.9820
% Monocytes	3.4–9.8%	Baseline	5.45 ± 1.56	5.77 ± 1.49 (*p* = 0.451)	5.94 ± 1.21 (*p* = 0.237)
		1-month	5.67 ± 1.65	6.08 ± 1.50 (*p* = 0.297)	5.82 ± 1.27 (*p* = 0.377)
		2-month	5.93 ± 2.00	5.92 ± 1.36 (*p* = 0.587)	6.03 ± 2.11 (*p* = 0.704)
% Eosinophils	0.7–9.2%	Baseline	3.20 ± 2.42	4.18 ± 2.31 (*p* = 0.095)	4.49 ± 0.95 (*p* = 0.226)
		1-month	3.11 ± 2.37	4.16 ± 2.36 * (*p* = 0.045)	4.51 ± 3.50 (*p* = 0.060)
		2-month	2.90 ± 1.96	3.88 ± 2.03 (*p* = 0.086)	4.46 ± 3.80 (*p* = 0.089)
% Basophils	0.0–2.6%	Baseline	0.64 ± 0.24	0.69 ± 0.22 (*p* = 0.356)	0.65 ± 0.29 (*p* = 0.920)
		1-month	0.67 ± 0.24	0.68 ± 0.27 (*p* = 0.819)	0.69 ± 0.32 (*p* = 0.710)
		2-month	0.73 ± 0.30	0.75 ± 0.24 (*p* = 0.668)	0.73 ± 0.49 (*p* = 0.403)

Data are reported as mean ± SD. * *p*-value < 0.05 compared with placebo. MPV: mean platelet volume, MCV: mean corpuscular volume, MCH: mean corpuscular hemoglobin, RDW: red cell distribution width, MCHC: mean corpuscular hemoglobin concentration.

**Table 12:** Clinical chemistry changes in participants consuming functional jelly drinks at baseline, 1 month, and 2 months.

Parameters	Reference	Time	Placebo (N = 25)	Low Dose (N = 23)	High Dose (N = 25)
Glucose	70–110 mg/dL	Baseline	97.72 ± 11.91	102.70 ± 9.54 (*p* = 0.134)	103.16 ± 12.30 (*p* = 0.095)
		1-month	97.80 ± 10.25	101.91 ± 17.33 (*p* = 0.620)	103.08 ± 12.56 (*p* = 0.203)
		2-month	96.24 ± 12.31	100.70 ± 6.90 (*p* = 0.077)	99.96 ± 12.95 (*p* = 0.377)
Blood urea nitrogen	5.8–19.1 mg/dL	Baseline	10.49 ± 2.94	11.27 ± 3.55 (*p* = 0.388)	10.46 ± 2.79 (*p* = 0.971)
		1-month	10.75 ± 3.20	10.71 ± 3.22 (*p* = 0.965)	10.28 ± 2.82 (*p* = 0.593)
		2-month	10.90 ± 2.74	10.74 ± 2.52 (*p* = 0.836)	10.06 ± 2.35 (*p* = 0.247)
Creatinine	0.5–1.5 mEq/L	Baseline	0.85 ± 0.20	0.93 ± 0.12 (*p* = 0.142)	0.85 ± 0.18 (*p* = 0.890)
		1-month	0.91 ± 0.20	0.93 ± 0.15 (*p* = 0.649)	0.86 ± 0.19 (*p* = 0.398)
		2-month	0.92 ± 0.21	0.95 ± 0.14 (*p* = 0.505)	0.87 ± 0.17 (*p* = 0.354)
Uric acid	2.7–7.0 mg/dL	Baseline	4.74 ± 1.20	5.47 ± 1.08 (*p* = 0.082)	4.94 ± 1.20 (*p* = 0.804)
		1-month	4.98 ± 1.25	5.34 ± 1.06 (*p* = 0.338)	5.21 ± 1.60 (*p* = 0.529)
		2-month	5.07 ± 1.15	5.42 ± 1.15 (*p* = 0.318)	5.02 ± 1.30 (*p* = 0.880)
Sodium	130–147 mEq/L	Baseline	137.96 ± 1.67	137.87 ± 1.60 (*p* = 0.836)	137.88 ± 1.20 (*p* = 0.851)
		1-month	138.92 ± 1.78	139.22 ± 1.54 (*p* = 0.522)	138.68 ± 1.46 (*p* = 0.598)
		2-month	138.80 ± 1.35	139.22 ± 1.73 (*p* = 0.358)	138.12 ± 1.59 (*p* = 0.128)
Potassium	3.4–4.7 mEq/L	Baseline	4.41 ± 0.32	4.29 ± 0.33 (*p* = 0.181)	4.31 ± 0.28 (*p* = 0.257)
		1-month	4.38 ± 0.35	4.39 ± 0.29 (*p* = 0.895)	4.26 ± 0.23 (*p* = 0.156)
		2-month	4.52 ± 0.09	4.38 ± 0.08 (*p* = 0.219)	4.37 ± 0.07 (*p* = 0.194)
Bicarbonate	20.6–28.3 mEq/L	Baseline	22.30 ± 2.82	21.64 ± 1.75 (*p* = 0.291)	22.06 ± 1.70 (*p* = 0.686)
		1-month	21.84 ± 2.23	21.70 ± 1.81 (*p* = 0.797)	21.79 ± 1.49 (*p* = 0.916)
		2-month	22.12 ± 2.25	22.01 ± 2.20 (*p* = 0.843)	22.06 ± 1.51 (*p* = 0.916)
Chloride	96–107 mEq/L	Baseline	101.04 ± 1.59	100.75 ± 1.77 (*p* = 0.511)	100.52 ± 2.02 (*p* = 0.312)
		1-month	101.44 ± 2.00	101.17 ± 1.99 (*p* = 0.625)	101.04 ± 1.62 (*p* = 0.454)
		2-month	101.20 ± 1.96	101.70 ± 2.03 (*p* = 0.366)	101.48 ± 1.66 (*p* = 0.601)
Protein	6.5–8.8 g/dL	Baseline	7.44 ± 0.31	7.65 ± 0.50 (*p* = 0.105)	7.48 ± 0.46 (*p* = 0.768)
		1-month	7.38 ± 0.40	7.59 ± 0.33 (*p* = 0.062)	7.31 ± 0.32 (*p* = 0.339)
		2-month	7.40 ± 0.39	7.53 ± 0.30 (*p* = 0.254)	7.33 ± 0.40 (*p* = 0.467)
Globulin	2.6–3.4 g/dL	Baseline	3.00 ± 0.31	3.01 ± 0.38 (*p* = 0.901)	2.91 ± 0.39 (*p* = 0.370)
		1-month	2.92 ± 0.29	3.00 ± 0.30 (*p* = 0.307)	2.83 ± 0.38 (*p* = 0.135)
		2-month	2.92 ± 0.34	2.97 ± 0.28 (*p* = 0.537)	2.85 ± 0.28 (*p* = 0.400)
Total bilirubin	0.3–1.5 mg/dL	Baseline	0.48 ± 0.26	0.60 ± 0.30 (*p* = 0.326)	0.69 ± 0.57 (*p* = 0.068)
		1-month	0.42 ± 0.24	0.54 ± 0.31 (*p* = 0.074)	0.68 ± 0.46 * (*p* = 0.011)
		2-month	0.52 ± 0.24	0.54 ± 0.33 (*p* = 0.731)	0.66 ± 0.31 (*p* = 0.474)
Direct bilirubin	0.0–0.5 mg/dL	Baseline	0.18 ± 0.08	0.23 ± 0.11 (*p* = 0.227)	0.25 ± 0.12 (*p* = 0.064)
		1-month	0.19 ± 0.10	0.20 ± 0.10 (*p* = 0.943)	0.23 ± 0.10 (*p* = 0.176)
		2-month	0.23 ± 0.10	0.19 ± 0.10 (*p* = 0.100)	0.21 ± 0.10 (*p* = 0.420)
ALT	4–36 U/L	Baseline	18.76 ± 12.05	18.26 ± 10.45 (*p* = 0.844)	19.32 ± 11.68 (*p* = 0.734)
		1-month	15.68 ± 8.07	18.70 ± 9.74 (*p* = 0.448)	19.88 ± 3.96 (*p* = 0.281)
		2-month	16.76 ± 10.49	19.30 ± 7.96 (*p* = 0.172)	17.23 ± 11.29 (*p* = 0.969)
AST	12–32 U/L	Baseline	20.28 ± 5.74	19.87 ± 5.31 (*p* = 0.901)	21.08 ± 7.25 (*p* = 0.697)
		1-month	19.92 ± 5.45	21.87 ± 10.48 (*p* = 0.725)	23.36 ± 4.23 (*p* = 0.838)
		2-month	21.28 ± 5.50	21.43 ± 5.15 (*p* = 0.934)	20.76 ± 8.24 (*p* = 0.777)
ALP	42–121 U/L	Baseline	65.42 ± 19.28	62.87 ± 12.80 (*p* = 0.636)	67.44 ± 21.56 (*p* = 0.701)
		1-month	69.04 ± 34.81	60.52 ± 11.43 (*p* = 0.233)	65.76 ± 20.39 (*p* = 0.637)
		2-month	70.16 ± 34.57	62.30 ± 11.43 (*p* = 0.249)	64.60 ± 16.79 (*p* = 0.404)
Amylase	25–125 U/L	Baseline	95.96 ± 27.36	90.09 ± 25.82 (*p* = 0.723)	78.36 ± 24.40 (*p* = 0.055)
		1-month	90.51 ± 28.75	89.71 ± 27.38 (*p* = 0.921)	94.01 ± 28.15 (*p* = 0.673)
		2-month	90.38 ± 14.15	87.77 ± 13.52 (*p* = 0.515)	92.36 ± 13.90 (*p* = 0.629)
LDH	0–250 U/L	Baseline	180.35 ± 28.50	166.50 ± 24.76 (*p* = 0.150)	170.12 ± 24.95 (*p* = 0.261)
		1-month	191.41 ± 41.15	180.29 ± 35.29 (*p* = 0.450)	181.56 ± 43.82 (*p* = 0.488)
		2-month	198.69 ± 49.52	178.27 ± 32.00 (*p* = 0.232)	167.82 ± 37.24 (*p* = 0.075)
CK	28–140 U/L	Baseline	99.53 ± 46.58	117.31 ± 43.99 (*p* = 0.143)	111.88 ± 71.67 (*p* = 0.863)
		1-month	120.76 ± 68.87	141.21 ± 99.30 (*p* = 0.722)	131.13 ± 64.65 (*p* = 0.748)
		2-month	107.36 ± 43.61	108.40 ± 54.70 (*p* = 0.963)	108.73 ± 53.79 (*p* = 0.950)
GGT	0–50 U/L	Baseline	30.36 ± 5.37	34.39 ± 7.89 (*p* = 0.369)	35.46 ± 7.85 (*p* = 0.787)
		1-month	28.04 ± 4.91	29.52 ± 4.46 (*p* = 0.160)	47.96 ± 17.78 (*p* = 0.885)
		2-month	28.20 ± 4.71	31.83 ± 4.36 (*p* = 0.124)	39.13 ± 12.18 (*p* = 0.869)
Homocysteine	0–15 U/L	Baseline	11.13 ± 2.76	12.48 ± 2.71 (*p* = 0.095)	10.88 ± 2.74 (*p* = 0.755)
		1-month	10.80 ± 3.52	11.13 ± 2.53 (*p* = 0.767)	11.08 ± 5.00 (*p* = 0.798)
		2-month	11.00 ± 3.09	11.43 ± 2.04 (*p* = 0.602)	10.72 ± 3.20 (*p* = 0.731)
Cholesterol	127–262 mg/dL	Baseline	203.36 ± 29.74	197.13 ± 30.23 (*p* = 0.497)	201.84 ± 34.51 (*p* = 0.865)
		1-month	212.68 ± 29.46	195.74 ± 34.96 (*p* = 0.064)	196.16 ± 29.04 (*p* = 0.065)
		2-month	210.04 ± 27.68	193.57 ± 26.40 (*p* = 0.051)	201.60 ± 31.74 (*p* = 0.303)
Triglyceride	10–200 mg/dL	Baseline	107.52 ± 49.39	108.30 ± 53.66 (*p* = 0.853)	92.96 ± 46.07 (*p* = 0.252)
		1-month	98.76 ± 37.31	97.83 ± 32.10 (*p* = 0.932)	92.40 ± 42.76 (*p* = 0.554)
		2-month	113.44 ± 58.35	111.35 ± 49.47 (*p* = 0.887)	101.60 ± 43.81 (*p* = 0.414)
HDL-C	>35 mg/dL	Baseline	63.60 ± 19.26	62.87 ± 17.18 (*p* = 0.875)	64.36 ± 10.12 (*p* = 0.867)
		1-month	65.80 ± 16.97	59.96 ± 15.07 (*p* = 0.204)	62.24 ± 9.29 (*p* = 0.861)
		2-month	66.12 ± 19.29	60.22 ± 14.38 (*p* = 0.189)	62.16 ± 11.43 (*p* = 0.367)
LDL-C	10–150 mg/dL	Baseline	128.56 ± 33.99	123.91 ± 26.25 (*p* = 0.608)	127.64 ± 32.40 (*p* = 0.917)
		1-month	136.80 ± 34.88	124.26 ± 28.95 (*p* = 0.159)	126.24 ± 27.02 (*p* = 0.225)
		2-month	132.28 ± 30.46	119.43 ± 20.78 (*p* = 0.112)	127.92 ± 30.03 (*p* = 0.579)

Data are reported as mean ± SD. * *p*-value < 0.05 compared with placebo. Reference value: Laboratory Unit, Srinagarind Hospital, Faculty of Medicine, Khon Kaen University. AST: aspartate aminotransferase; ALT: alanine aminotransferase; ALP: alkaline phosphatase; LDH: lactate dehydrogenase, CK: creatine kinase, GGT: gamma-glutamyl transferase; HDL-C: high-density lipoprotein cholesterol; LDL-C: low-density lipoprotein cholesterol.

## Data Availability

The data presented in this study are available on request from the corresponding author. The data are not publicly available due to trade secrecy and the ongoing patent registration process.

## Supplementary Materials

The following supporting information can be downloaded at: https://www.mdpi.com/article/10.3390/foods15142461/s1, File S1: physical activity of participants; File S2: Food Consumption of participants measured by using Food Frequency Questionnaire (FFQ).

## References

[1] International Labour Organization (2022). World Employment and Social Outlook: Trends 2022. https://www.ilo.org/global/research/global-reports/weso/trends2022/WCMS_834081/lang--en/index.htm.

[2] de Krijger E., Ten Klooster P.M., Geuze E., Kelders S.M., Bohlmeijer E.T. (2025). Work-Stressors and Depression and Anxiety-A Longitudinal Study of the Moderating Role of Self-Compassion. Stress Health.

[3] Mahmud S., Mohsin M., Dewan M.N., Muyeed A. (2023). The Global Prevalence of Depression, Anxiety, Stress, and Insomnia Among General Population During COVID-19 Pandemic: A Systematic Review and Meta-analysis. Trends Psychol..

[4] World Health Organization (2025). Over a Billion People Living with Mental Health Conditions–Services Require Urgent Scale-Up. https://www.who.int/news/item/02-09-2025-over-a-billion-people-living-with-mental-health-conditions-services-require-urgent-scale-up.

[5] Carvalho C., Reis C., Serrano M. (2025). Exploring the Link Between Stress and Working Memory in Adults. Int. J. Env. Res. Public Health.

[6] Giorgi G., Lecca L.I., Leon-Perez J.M., Pignata S., Topa G., Mucci N. (2020). Emerging Issues in Occupational Disease: Mental Health in the Aging Working Population and Cognitive Impairment-A Narrative Review. BioMed Res. Int..

[7] Luethi M., Meier B., Sandi C. (2009). Stress effects on working memory, explicit memory, and implicit memory for neutral and emotional stimuli in healthy men. Front. Behav. Neurosci..

[8] Friedel E., Sebold M., Kuitunen-Paul S., Nebe S., Veer I.M., Zimmermann U.S., Schlagenhauf F., Smolka M.N., Rapp M., Walter H. (2017). How Accumulated Real Life Stress Experience and Cognitive Speed Interact on Decision-Making Processes. Front. Hum. Neurosci..

[9] Wright E.C. (2020). Neurobiological Insights Into Stress-Induced Attention Deficit. Biol. Psychiatry.

[10] Kim H., Park Y.S., Kim S.H., Hurh K., Kim J., Park E.C., Jang S.I. (2023). Association between stress-related disorders and the risk of dementia using the Korean National Sample Cohort: A matched cohort study. Sci. Rep..

[11] Alzheimer’s Society The Economic Impact of Dementia. https://www.alzheimers.org.uk/what-we-do/policy-and-influencing/economic-impact-of-dementia.

[12] Galanakis C.M. (2021). Functionality of Food Components and Emerging Technologies. Foods.

[13] Ferat-Osorio E., Maldonado-García J.L., Pavón L. (2024). How inflammation influences psychiatric disease. World J. Psychiatry.

[14] Franzoni F., Scarfò G., Guidotti S., Fusi J., Asomov M., Pruneti C. (2021). Oxidative Stress and Cognitive Decline: The Neuroprotective Role of Natural Antioxidants. Front. Neurosci..

[15] Kocamer Şahin Ş., Aslan E. (2024). Inflammation as a Neurobiological Mechanism of Cognitive Impairment in Psychological Stress. J. Integr. Neurosci..

[16] Salim S. (2014). Oxidative stress and psychological disorders. Curr. Neuropharmacol..

[17] Vollbracht C., Werner M. (2024). The role of inflammation and oxidative stress in the pathophysiology of depressions: Time to consider vitamin C deficiency. Explor. Neurosci..

[18] Gęgotek A., Skrzydlewska E. (2022). Antioxidative and Anti-Inflammatory Activity of Ascorbic Acid. Antioxidants.

[19] Hunt T., Pontifex M.G., Vauzour D. (2024). (Poly)phenols and brain health-beyond their antioxidant capacity. FEBS Lett..

[20] Musa K.H., Abdullah A., Subramainiam V. (2015). Flavonoid Profile and Antioxidant Activity of Pink Guava. Sci. Asia.

[21] Nguyen H.-H., Wattanathorn J., Thukham-Mee W., Muchimapura S., Paholpak P. (2025). Development of a Guava Jelly Drink with Potential Antioxidant, Anti-Inflammation, Neurotransmitter, and Gut Microbiota Benefits. Foods.

[22] Bhattacharya S., Montag D. (2015). Acetylcholinesterase inhibitor modifications: A promising strategy to delay the progression of Alzheimer’s disease. Neural Regen. Res..

[23] Zuin M., Cherubini A., Volpato S., Ferrucci L., Zuliani G. (2022). Acetyl-cholinesterase-inhibitors slow cognitive decline and decrease overall mortality in older patients with dementia. Sci. Rep..

[24] Peth-Nui T., Wattanathorn J., Muchimapura S., Tong-Un T., Piyavhatkul N., Rangseekajee P., Ingkaninan K., Vittaya-Areekul S. (2012). Effects of 12-Week Bacopa monnieri Consumption on Attention, Cognitive Processing, Working Memory, and Functions of Both Cholinergic and Monoaminergic Systems in Healthy Elderly Volunteers. Evid.-Based Complement. Altern. Med..

[25] Kennedy D.O., Scholey A.B., Drewery L., Marsh V.R., Moore B., Ashton H. (2003). Electroencephalograph effects of single doses of Ginkgo biloba and Panax ginseng in healthy young volunteers. Pharmacol. Biochem. Behav..

[26] Luck S.J., Luck S.J. (2005). An introduction to event-related potentials and their neural origins. An Introduction to the Event-Related Potential Technique.

[27] Drake M.E., Pakalnis A., Padamadan H. (1989). Long-latency auditory event related potentials in migraine. Headache.

[28] Sur S., Sinha V.K. (2009). Event-related potential: An overview. Indian J. Psychiatry.

[29] Moss M.C., Scholey A.B., Wesnes K. (1998). Oxygen administration selectively enhances cognitive performance in healthy young adults: A placebo-controlled double-blind crossover study. Psychopharmacology.

[30] Wesnes K.A. (2001). The use of cognitive tests to facilitate drug and dose selection in Phase I and to optimise dosing in Phase IV. Int. Congr. Ser..

[31] Wattanathorn J., Somboonporn W., Thukham-Mee W., Sungkamnee S. (2022). Memory-Enhancing Effect of 8-Week Consumption of the Quercetin-Enriched Culinary Herbs-Derived Functional Ingredients: A Randomized, Double-Blind, Placebo-Controlled Clinical Trial. Foods.

[32] Wattanathorn J., Thukham-Mee W., Tong-Un T., Sangartit W., Somboonporn W., Paholpak P. (2025). A Randomized, Double-Blind, Placebo-Controlled, Parallel-Group, 8-Week Pilot Study of Tuna-Byproduct-Derived Novel Supplements for Managing Cellular Senescence and Cognitive Decline in Perimenopausal and Postmenopausal Women. Antioxidants.

[33] Wattanathorn J., Mator L., Muchimapura S., Tongun T., Pasuriwong O., Piyawatkul N., Yimtae K., Sripanidkulchai B., Singkhoraard J. (2008). Positive modulation of cognition and mood in the healthy elderly volunteer following the administration of Centella asiatica. J. Ethnopharmacol..

[34] Cohen S., Kamarck T., Mermelstein R. (1983). A global measure of perceived stress. J. Health Soc. Behav..

[35] Makhubela M. (2022). Assessing psychological stress in South African university students: Measurement validity of the perceived stress scale (PSS-10) in diverse populations. Curr. Psychol..

[36] Wu Y., Levis B., Sun Y., He C., Krishnan A., Neupane D., Bhandari P.M., Negeri Z., Benedetti A., Thombs B.D. (2021). DEPRESsion Screening Data (DEPRESSD) HADS Group. Accuracy of the Hospital Anxiety and Depression Scale Depression subscale (HADS-D) to screen for major depression: Systematic review and individual participant data meta-analysis. BMJ..

[37] Lloyd M., Sugden N., Thomas M., McGrath A., Skilbeck C. (2023). The structure of the Hospital Anxiety and Depression Scale: Theoretical and methodological considerations. Br. J. Psychol..

[38] Finberg J.P., Rabey J.M. (2016). Inhibitors of MAO-A and MAO-B in Psychiatry and Neurology. Front. Pharmacol..

[39] Murwidi I.C. (2018). Pengaruh Kerja Shift terhadap Kadar Glutamic Acid Decarboxylase 65 (GAD65) dalam Serum Darah. J. Kesehat..

[40] Ohkawa H., Ohishi N., Yagi K. (1979). Assay for lipid peroxides in animal tissues by thiobarbituric acid reaction. Anal. Biochem..

[41] Wattanathorn J., Ohnon W., Thukhammee W., Muchmapura S., Wannanon P., Tong-Un T. (2019). Cerebroprotective Effect against Cerebral Ischemia of the Combined Extract of Oryza sativa and Anethum graveolens in Metabolic Syndrome Rats. Oxidative Med. Cell. Longev..

[42] Wattanathorn J., Tong-un T., Thukham-mee W., Paholpak P., Rangseekhajee P. (2023). A Randomized, Double-Blind, Placebo-Controlled Study of an Anthocyanin-Rich Functional Ingredient on Cognitive Function and Eye Dryness in Late Adulthood Volunteers: Roles of Epigenetic and Gut Microbiome Modulations. Nutrients.

[43] Filippone A., Barbieri U., Corbo M.R., Sinigaglia M., Bivilacqua A. (2025). The Gut–Brain Axis and Probiotics in Beverages and Liquid Preparations: A PRISMA Systematic Review on Cognitive Function Enhancement. Beverages.

[44] Thornton A.R., Harmer M., Lavoie B.A. (2007). Selective attention increases the temporal precision of the auditory N100 event-related potential. Hear. Res..

[45] Diamond A. (2013). Executive functions. Annu. Rev. Psychol..

[46] Grinspun N., Nijs L., Kausel L., Onderdijk K., Sepúlveda N., Rivera-Hutinel A. (2020). Selective Attention and Inhibitory Control of Attention Are Correlated With Music Audiation. Front. Psychol..

[47] Lin G.H., Bai D., Huang Y.J., Lee S.C., Vu M.T.T., Chiu T.H. (2025). Artificial Intelligence-Based Computerized Digit Vigilance Test in Community-Dwelling Older Adults: Development and Validation Study. JMIR Med. Inform..

[48] Lanska M., Olds J.M., Westerman D.L. (2014). Fluency effects in recognition memory: Are perceptual fluency and conceptual fluency interchangeable?. J. Exp. Psychol. Learn. Mem. Cogn..

[49] Corriveau A., Chao A.F., de Bettencourt M.T., Rosenberg M.D. (2025). Recognition memory fluctuates with sustained attention regardless of task relevance. Psychon. Bull. Rev..

[50] Pavarini S.C.I., Brigola A.G., Luchesi B.M., Souza É.N., Rossetti E.S., Fraga F.J., Guarisco L.P.C., Terassi M., Oliveira N.A., Hortense P. (2018). On the use of the P300 as a tool for cognitive processing assessment in healthy aging: A review. Dement. Neuropsychol..

[51] Zigmond A.S., Snaith R.P. (1983). The hospital anxiety and depression scale. Acta Psychiatr. Scand..

[52] Trofin D.M., Sardaru D.P., Trofin D., Onu I., Tutu A., Onu A., Onită C., Galaction A.I., Matei D.V. (2025). Oxidative Stress in Brain Function. Antioxidants.

[53] Singh P., Barman B., Thakur M.K. (2022). Oxidative stress-mediated memory impairment during aging and its therapeutic intervention by natural bioactive compounds. Front. Aging Neurosci..

[54] Bouayed J., Rammal H., Soulimani R. (2009). Oxidative stress and anxiety: Relationship and cellular pathways. Oxidative Med. Cell. Longev..

[55] Correia A.S., Cardoso A., Vale N. (2023). Oxidative Stress in Depression: The Link with the Stress Response, Neuroinflammation, Serotonin, Neurogenesis and Synaptic Plasticity. Antioxidants.

[56] Kim E., Zhao Z., Rzasa J.R., Glassman M., Bentley W.E., Chen S., Kelly D.L., Payne G.F. (2021). Association of acute psychosocial stress with oxidative stress: Evidence from serum analysis. Redox Biol..

[57] Mekhora C., Lamport D.J., Spencer J.P.E. (2024). An overview of the relationship between inflammation and cognitive function in humans, molecular pathways and the impact of nutraceuticals. Neurochem. Int..

[58] Harrington E.E., Graham-Engeland J.E., Lipton R.B., Roque N.A., Sliwinski M.J., Engeland C.G. (2023). Inflammation and working memory performance in everyday life: Gender differences among older adults with and without cognitive impairment. Alzheimer’s Dement..

[59] Liu Y.Z., Wang Y.X., Jiang C.L. (2017). Inflammation: The Common Pathway of Stress-Related Diseases. Front. Hum. Neurosci..

[60] Giollabhui N.M., Slaney C., Hemani G., Foley É.M., van der Most P.J., Nolte I.M., Snieder H., Smith G.D., Khandaker G., Hartman C.A. (2025). Role of Inflammation in Depressive and Anxiety Disorders, Affect, and Cognition: Genetic and Non-Genetic Findings in the Lifelines Cohort Study. Transl. Psychiatry.

[61] Anders M. (2025). Neurotransmitters and Their Influence on Behavior and Cognition. Neuropsychiatry.

[62] Xu X., Zhao H., Song Y., Cai H., Zhao W., Tang J., Zhu J., Yu Y. (2024). Molecular mechanisms underlying the neural correlates of working memory. BMC Biol..

[63] Kao C.H. (2024). Neurotransmitters and Their Influence on Mental Health Disorders. Neurosci. Psych. Open Access..

[64] Mora F., Segovia G., Del Arco A., de Blas M., Garrido P. (2012). Stress, neurotransmitters, corticosterone and body-brain integration. Brain Res..

[65] Pisani A., Paciello F., Del Vecchio V., Malesci R., De Corso E., Cantone E., Fetoni A.R. (2023). The Role of BDNF as a Biomarker in Cognitive and Sensory Neurodegeneration. J. Pers. Med..

[66] Miranda M., Morici J.F., Zanoni M.B., Bekinschtein P. (2019). Brain-Derived Neurotrophic Factor: A Key Molecule for Memory in the Healthy and the Pathological Brain. Front. Cell. Neurosci..

[67] Miao Z., Wang Y., Sun Z. (2020). The Relationships Between Stress, Mental Disorders, and Epigenetic Regulation of BDNF. Int. J. Mol. Sci..

[68] Echouffo-Tcheugui J.B., Conner S.C., Himali J.J., Maillard P., DeCarli C.S., Beiser A.S., Vasan R.S., Seshadri S. (2018). Circulating cortisol and cognitive and structural brain measures: The Framingham Heart Study. Neurology.

[69] Sherman B.E., Harris B.B., Turk-Browne N.B., Sinha R., Goldfarb E.V. (2023). Hippocampal Mechanisms Support Cortisol-Induced Memory Enhancements. J. Neurosci..

[70] Tooley K.L. (2020). Effects of the Human Gut Microbiota on Cognitive Performance, Brain Structure and Function: A Narrative Review. Nutrients.

[71] Kuijer E.J., Steenbergen L. (2023). The microbiota-gut-brain axis in hippocampus-dependent learning and memory: Current state and future challenges. Neurosci. Biobehav Rev..

[72] Sudo N. (2019). Role of gut microbiota in brain function and stress-related pathology. Biosci. Microbiota Food Health.

[73] Xiong R.G., Li J., Cheng J., Zhou D.D., Wu S.X., Huang S.Y., Saimaiti A., Yang Z.J., Gan R.Y., Li H.B. (2023). The Role of Gut Microbiota in Anxiety, Depression, and Other Mental Disorders as Well as the Protective Effects of Dietary Components. Nutrients.

[74] Zimmerman C.N., Wong R.Y., Dijkstra P.D. (2026). Is oxidative stress in the brain correlated with plasma markers of oxidative stress?. Comp. Biochem. Physiol. Part A Mol. Integr. Physiol..

[75] Ahrițculesei R.V., Boldeanu L., Assani M.Z., Mitrea A., Obleaga C.V., Vladu I.M., Clenciu D., Boldeanu M.V., Vere C.C. (2025). Neurotransmitter Levels (Dopamine, Epinephrine, Norepinephrine, Serotonin) and Associations with Lipid Profiles in Patients with Prediabetes or Newly Diagnosed Type 2 Diabetes Mellitus. Int. J. Mol. Sci..

[76] Warren K.N., Beason-Held L.L., Carlson O., Egan J.M., An Y., Doshi J., Davatzikos C., Ferrucci L., Resnick S.M. (2018). Elevated Markers of Inflammation Are Associated With Longitudinal Changes in Brain Function in Older Adults. J. Gerontol. A Biol. Sci. Med. Sci..

[77] Hou C., Hsieh C.J., Li S., Lee H., Graham T.J., Xu K., Weng C.C., Doot R.K., Chu W., Chakraborty S.K. (2018). Development of a Positron Emission Tomography Radiotracer for Imaging Elevated Levels of Superoxide in Neuroinflammation. ACS Chem. Neurosci..

[78] Park E., Gallezot J.D., Delgadillo A., Liu S., Planeta B., Lin S.F., O’Connor K.C., Lim K., Lee J.Y., Chastre A. (2015). (11)C-PBR28 imaging in multiple sclerosis patients and healthy controls: Test-retest reproducibility and focal visualization of active white matter areas. Eur. J. Nucl. Med. Mol. Imaging.

[79] Kobayashi M., Jiang T., Telu S., Zoghbi S.S., Gunn R.N., Rabiner E.A., Owen D.R., Guo Q., Pike V.W., Innis R.B. (2018). 11C-DPA-713 has much greater specific binding to translocator protein 18 kDa (TSPO) in human brain than 11C-( R)-PK11195. J. Cereb. Blood Flow Metab..

[80] Winkeler A., Boisgard R., Awde A.R., Dubois A., Thézé B., Zheng J., Ciobanu L., Dollé F., Viel T., Jacobs A.H. (2012). The translocator protein ligand [¹⁸F]DPA-714 images glioma and activated microglia in vivo. Eur. J. Nucl. Med. Mol. Imaging.

[81] Placzek M.S., Zhao W., Wey H.Y., Morin T.M., Hooker J.M. (2016). PET Neurochemical Imaging Modes. Semin. Nucl. Med..

[82] Misera A., Marlicz W., Podkówka A., Łoniewski I., Skonieczna-Żydecka K. (2024). Possible application of Akkermansia muciniphila in stress management. Microbiome Res. Rep..

[83] Maftoon H., Davar Siadat S., Tarashi S., Soroush E., Basir Asefi M., Rahimi Foroushani A., Mehdi Soltan Dallal M. (2024). Ameliorative effects of Akkermansia muciniphila on anxiety-like behavior and cognitive deficits in a rat model of Alzheimer’s disease. Brain Res..

[84] Li N., Tan S., Wang Y., Deng J., Wang N., Zhu S., Tian W., Xu J., Wang Q. (2023). Akkermansia muciniphila supplementation prevents cognitive impairment in sleep-deprived mice by modulating microglial engulfment of synapses. Gut Microbes.

[85] Kupcova I., Danisovic L., Grgac I., Harsanyi S. (2022). Anxiety and Depression: What Do We Know of Neuropeptides?. Behav. Sci..

[86] Zhao P., Qian X., Nie Y., Sun N., Wang Z., Wu J., Wei C., Ma R., Wang Z., Chai G. (2019). Neuropeptide S Ameliorates Cognitive Impairment of APP/PS1 Transgenic Mice by Promoting Synaptic Plasticity and Reducing Aβ Deposition. Front. Behav. Neurosci..

[87] Okamura N., Reinscheid R.K. (2007). Neuropeptide S: A novel modulator of stress and arousal: Review. Stress.

[88] Schmidt-Wilcke T., Fuchs E., Funke K., Vlachos A., Müller-Dahlhaus F., Puts N.A.J., Harris R.E., Edden R.A.E. (2018). GABA-from Inhibition to Cognition: Emerging Concepts. Neuroscientist.

[89] Goddard A.W. (2016). Cortical and subcortical gamma amino acid butyric acid deficits in anxiety and stress disorders: Clinical implications. World J. Psychiatry.

[90] Tınok A.A., Karabay A., Jong J., Balta G., Akyürek E.G. (2023). Effects of gamma-aminobutyric acid on working memory and attention: A randomized, double-blinded, placebo-controlled, crossover trial. J. Psychopharmacol..

[91] Singh B., Bennett H., Miatke A., Dumuid D., Curtis R., Ferguson T., Brinsley J., Szeto K., Petersen J.M., Gough C. (2025). Effectiveness of exercise for improving cognition, memory and executive function: A systematic umbrella review and meta-meta-analysis. Br. J. Sports Med..

[92] Puri S., Shaheen M., Grover B. (2023). Nutrition and cognitive health: A life course approach. Front. Public Health.

