# Supplementary material for: A Pilot Randomized, Double-Blind, Placebo-Controlled Parallel Group Trial Evaluating the Effect of 8 Week-Consumption of Guava Jelly Drink in Improving Cognition and Mental Well-Being in Working-Age Adults
Source: Foods. 2026 Jul 11;15(14):2461. doi: 10.3390/foods15142461 (PMC13409673; doi:10.3390/foods15142461)
Supplement: Supplementary file 1 [file foods-15-02461-s001.zip › File S1.pdf]

Supplementary Material S1. The physical activities of participants who consumed functional jelly drinks at baseline, 1-month, and 2-month. Data were reported as mean ± SD.

Parameters		Baseline		
		Placebo (N=25)	Low dose (N=23)	High dose (N=25)
Strenuous Activities	Fast running	0.56±0.27	0.57±0.33 (p=0.652)	0.56±0.23 (p=0.801)
	Heavy lifting	0.16±0.11	0.78±0.43 (p=0.297)	0.88±0.40 (p=0.114)
	Exercise (minutes)	41.40±12.58	32.87±10.17 (p=0.973)	66.04±21.67 (p=0.787)
Moderate Strength Activities	Cycling on flat ground	0.56±0.31	0.22±0.18 (p=0.429)	2.68±2.40 (p=0.736)
	Light Lifting	0.52±0.24	0.74±0.40 (p=0.705)	1.20±0.44 (p=0.513)
Walking	Fast walking	3.80±0.61	2.96±0.64 (p=0.303)	3.60±0.59 (p=0.778)
	Slow walking	4.44±0.56	3.87±0.67 (p=0.689)	3.92±0.62 (p=0.642)
	Walking at home	3.88±0.59	2.57±0.56 (p=0.114)	4.00±0.59 (p=0.976)
Sitting	Sit at the desk	4.72±0.51	4.43±0.59 (p=0.906)	4.52±0.50 (p=0.680)
	Sit and chat with friends	3.80±0.59	2.70±0.59 (p=0.316)	3.92±0.59 (p=0.823)
	Sit and relax	4.08±0.59	2.70±0.59 (p=0.090)	3.92±0.59 (p=0.601)
	Sit and read a book	0.80±0.37	1.04±0.37 (p=0.433)	1.48±0.47 (p=0.153)
Other activities		0.00±0.00	0.00±0.00 (p=1.000)	0.00±0.00 (p=1.000)

Supplementary Material S1. The physical activities of participants who consumed functional jelly drinks at baseline, 1-month, and 2-month (Cont.)

Parameters		1-month		
		Placebo (N=25)	Low dose (N=23)	High dose (N=25)
Strenuous Activities	Fast running	0.56±0.28	0.87±0.34 (p=0.537)	0.64±0.35 (p=0.783)
	Heavy lifting	0.88±0.37	1.74±0.53 (p=0.205)	0.76±0.28 (p=0.980)
	Exercise (minutes)	73.80±19.71	25.87±7.69 (p=0.185)	93.80±24.46 (p=0.604)
Moderate Strength Activities	Cycling on flat ground	0.16±0.12	0.43±0.25 (p=0.323)	0.48±0.26 (p=0.362)
	Light Lifting	1.96±0.56	2.13±0.60 (p=0.972)	2.08±0.57 (p=0.837)
Walking	Fast walking	2.44±0.64	0.65±0.61 (p=0.536)	2.52±0.57 (p=0.707)
	Slow walking	3.76±0.64	3.78±0.64 (p=0.879)	2.56±0.62 (p=0.165)
	Walking at home	3.68±0.61	4.17±0.61 (p=0.531)	3.32±0.60 (p=0.756)
Sitting	Sit at the desk	4.40±0.54	4.22±0.58 (p=0.799)	4.84±0.52 (p=0.533)
	Sit and chat with friends	3.88±0.60	4.17±0.60 (p=0.898)	3.04±0.55 (p=0.291)
	Sit and relax	3.52±0.64	3.96±0.64 (p=0.812)	3.12±0.55 (p=0.689)
	Sit and read a book	1.04±0.42	0.91±0.37 (p=0.709)	1.00±0.39 (p=0.800)
Other activities		0.12±0.12	0.00±0.00 (p=0.337)	0.00±0.00 (p=0.317)

Supplementary Material S1. The physical activities of participants who consumed functional jelly drinks at baseline, 1-month, and 2-month (Cont.)

Parameters		2-month		
		Placebo (N=25)	Low dose (N=23)	High dose (N=25)
Strenuous Activities	Fast running	0.60±0.31	0.83±0.34 (p=0.217)	1.12±0.37 (p=0.063)
	Heavy lifting	0.92±0.36	1.22±0.45 (p=0.606)	1.04±0.34 (p=0.497)
	Exercise (minutes)	88.60±29.57	25.22±9.28 (p=0.143)	83.48±24.18 (p=0.765)
Moderate Strength Activities	Cycling on flat ground	0.08±0.08	3.87±2.70 (p=0.059)	0.48±0.27 (p=0.274)
	Light Lifting	2.56±0.65	2.43±0.62 (p=0.699)	2.40±0.57 (p=0.873)
Walking	Fast walking	3.44±0.67	2.78±0.62 (p=0.481)	2.36±0.56 (p=0.211)
	Slow walking	4.40±0.64	3.65±0.66 (p=0.393)	3.40±0.61 (p=0.311)
	Walking at home	3.60±0.67	2.78±0.60 (p=0.557)	3.96±0.60 (p=0.674)
Sitting	Sit at the desk	4.44±0.55	4.87±0.56 (p=0.720)	4.84±0.52 (p=0.792)
	Sit and chat with friends	3.12±0.63	3.30±0.63 (p=0.772)	3.12±0.56 (p=0.834)
	Sit and relax	3.80±0.61	2.87±0.59 (p=0.262)	2.96±0.58 (p=0.348)
	Sit and read a book	1.76±0.54	0.91±0.43 (p=0.257)	0.92±0.41 (p=0.222)
Other activities		0.04±0.04	0.00±0.00 (p=0.337)	0.00±0.00 (p=0.317)
