# Supplementary material for: A Pilot Randomized, Double-Blind, Placebo-Controlled Parallel Group Trial Evaluating the Effect of 8 Week-Consumption of Guava Jelly Drink in Improving Cognition and Mental Well-Being in Working-Age Adults
Source: Foods. 2026 Jul 11;15(14):2461. doi: 10.3390/foods15142461 (PMC13409673; doi:10.3390/foods15142461)
Supplement: Supplementary file 1 [file foods-15-02461-s001.zip › File S2.pdf]

Supplementary Material S2. Food frequency questionnaire of participants who consumed functional jelly drinks. Data were reported as mean ± SD.

Parameters	Baseline		
	Placebo (N=25)	Low dose (N=23)	High dose (N=25)
White rice (ladle)	21.04±2.20	22.89±2.75 (p=0.901)	20.98±2.46 (p=0.801)
Meat (piece)	56.12±10.88	71.39±13.44 (p=0.591)	78.86±13.14 (p=0.099)
Egg (egg)	9.56±1.62	7.83±1.39 (p=0.528)	9.88±1.62 (p=0.923)
Milk (glass/carton)	5.76±1.17	4.65±1.15 (p=0.495)	5.04±1.05 (p=0.738)
Vegetables (cup)	7.04±1.16	9.22±1.26 (p=0.242)	9.04±1.20 (p=0.243)
Fruits (cup)	4.12±1.09	4.96±1.15 (p=0.489)	4.10±0.89 (p=0.708)
Yogurt/fermented milk (cup)	0.88±0.22	1.22±0.23 (p=0.276)	1.04±0.24 (p=0.646)

Supplementary Material S2. Food frequency questionnaire of participants who consumed functional jelly drinks (Cont.)

Parameters	1-month		
	Placebo (N=25)	Low dose (N=23)	High dose (N=25)
White rice (ladle)	21.90±2.30	23.46±2.20 (p=0.584)	22.20±2.47 (p=0.984)
Meat (piece)	65.18±16.74	54.74±10.55 (p=0.910)	67.20±10.62 (p=0.273)
Egg (egg)	10.84±1.88	9.00±1.22 (p=0.951)	10.84±1.67 (p=0.756)
Milk (glass/carton)	5.24±1.11	4.09±0.69 (p=0.739)	4.58±0.86 (p=0.961)
Vegetables (cup)	7.94±1.30	9.04±1.17 (p=0.475)	10.64±1.33 (p=0.159)
Fruits (cup)	4.56±0.95	6.83±1.34 (p=0.205)	5.56±0.98 (p=0.418)
Yogurt/fermented milk (cup)	1.24±0.43	1.30±0.40 (p=0.821)	1.36±0.49 (p=0.923)

Supplementary Material S2. Food frequency questionnaire of participants who consumed functional jelly drinks (Cont.)

Parameters	2-month		
	**Placebo (N=25)**	**Low dose (N=23)**	**High dose (N=25)**
White rice (ladle)	21.08±2.40	20.65±1.19 (p=0.575)	20.74±2.37 (p=0.915)
Meat (piece)	72.40±17.62	58.78±11.73 (p=0.869)	76.76±10.51 (p=0.187)
Egg (egg)	9.80±1.48	10.22±1.60 (p=0.893)	11.84±1.74 (p=0.472)
Milk (glass/carton)	4.62±1.18	5.57±1.17 (p=0.413)	5.40±0.80 (p=0.196)
Vegetables (cup)	10.14±1.78	9.87±1.15 (p=0.679)	10.22±1.73 (p=0.938)
Fruits (cup)	4.88±1.19	6.48±1.34 (p=0.236)	4.02±0.74 (p=0.860)
Yogurt/fermented milk (cup)	1.20±0.34	2.00±0.76 (p=0.723)	1.20±0.31 (p=0.941)
